# The cumulative niche approach: A framework to assess the performance of ecological niche model projections

**DOI:** 10.1002/ece3.11060

**Published:** 2024-02-21

**Authors:** Eduardo Arlé, Tiffany Marie Knight, Marina Jiménez‐Muñoz, Dino Biancolini, Jonathan Belmaker, Carsten Meyer

**Affiliations:** ^1^ German Centre for Integrative Biodiversity Research (iDiv), Halle‐Jena‐Leipzig Leipzig Germany; ^2^ School of Zoology, George S. Wise Faculty of Life Sciences Tel Aviv University Tel Aviv Israel; ^3^ Department Community Ecology Helmholtz Centre for Environmental Research‐UFZ Halle (Saale) Germany; ^4^ Institute of Biology Martin Luther University Halle‐Wittenberg Halle (Saale) Germany; ^5^ Core Facility Statistical Consulting, Helmholz Centre Munich German Research Centre for Environmental Health GmbH Munich Germany; ^6^ Institute for BioEconomy (CNR‐IBE) National Research Council of Italy Rome Italy; ^7^ The Steinhardt Museum of Natural History Tel Aviv University Tel Aviv Israel; ^8^ Faculty of Biosciences, Pharmacy and Psychology University of Leipzig Leipzig Germany; ^9^ Faculty of Natural Sciences III – Agricultural and Nutritional Sciences, Geosciences and Computer Science Halle (Saale) Germany

**Keywords:** alien species, climate change, ecological modelling, ecological niche models, invasion biology, model projections, niche calculation, species distribution modelling, species range

## Abstract

Ecological Niche Models (ENMs) are often used to project species distributions within alien ranges and in future climatic scenarios. However, ENMs depend on species‐environment equilibrium, which may be absent for actively expanding species. We present a novel framework to estimate whether species have reached environmental equilibrium in their native and alien ranges. The method is based on the estimation of niche breadth with the accumulation of species occurrences. An asymptote will indicate exhaustive knowledge of the realised niches. We demonstrate the CNA framework for 26 species of mammals, amphibians, and birds. Possible outcomes of the framework include: (1) There is enough data to quantify the native and alien realised niches, allowing us to calculate niche expansion between the native and alien ranges, also indicating that ENMs can be reliably projected to new environmental conditions. (2) The data in the native range is not adequate but an asymptote is reached in the alien realised niche, indicating low confidence in our ability to evaluate niche expansion in the alien range but high confidence in model projections to new environmental conditions within the alien range. (3) There is enough data to quantify the native realised niche, but not enough knowledge about the alien realised niche, hindering the reliability of projections beyond sampled conditions. (4) Both the native and alien ranges do not reach an asymptote, and thus few robust conclusions about the species’ niche or future projections can be made. Our framework can be used to detect species’ environmental equilibrium in both the native and alien ranges, to quantify changes in the realised niche during the invasion processes, and to estimate the likely accuracy of model projections to new environmental conditions.

## Graphical abstract

The Cumulative Niche Approach: a novel framework to estimate the predictive power of Ecological Niche Model projections. Our theoretical framework allows to quantify changes in the realised niche during invasion processes and to estimate the likely accuracy of model projections to new environmental conditions.

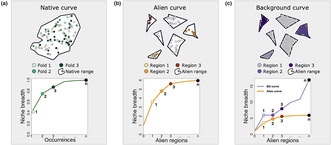

## 1 INTRODUCTION

Alien species and climate change are amongst the main causes of species extinctions (Bellard et al., 2016; Clavero & García‐Berthou, 2005; Urban, 2018). Despite a growing body of literature on biological invasions and efforts to prevent their spread (Schwindt et al., 2023), alien species are still expanding their distribution worldwide (Seebens et al., 2017). Previous research has suggested that climate change might create more favourable conditions for alien species, allowing them to expand their ranges and increase population sizes (Bellard et al., 2018; Walther et al., 2009). In contrast, climate change will act as a threat to many native species (Pacifici et al., 2015). To preserve native biodiversity against the combined impacts of climate change and biological invasions, it is urgent to understand which factors explain alien species' current distributions and to identify which areas could be suitable for them in the future (McGeoch & Jetz, 2019; Pyšek et al., 2020).

Ecological Niche Models (ENMs) are a powerful tool to characterise species' niches and quantify their ecological requirements (Srivastava et al., 2019). Most ENMs adopt a correlative approach, using the conditions found in species' localities of occurrence to infer their ecological niches (Elith & Leathwick, 2009; Zurell et al., 2020). ENMs are highly versatile and have been applied to a wide range of research questions in conservation biology, agriculture, ecology and evolution (Zurell et al., 2020), including forecasting how climate change will alter species' distributions (Sierra‐Morales et al., 2021) and predicting species' environmental suitabilities outside their native ranges (Cucco et al., 2021; Ringani et al., 2022). ENMs assume that the species currently occurs in all suitable environments (i.e. is at a species‐environment equilibrium; Foster et al., 2022). However, the current occupancies of many species do not reflect all the environments that are suitable, and in these cases, the ENM model will underestimate the species' possible realised niche will not accurately project its suitability to new areas or future climatic projections (Liu et al., 2020; Ludwig et al., 2023; Manzoor et al., 2018; Parravicini et al., 2015; Werkowska et al., 2017). For example, many species have often been shown to occupy novel environments in their alien ranges (Hui, 2022; Liu et al., 2020) and thus ENMs created using occupancies from the native range would not have projected the alien to occur in those environments.

To make robust and reliable projections of alien ranges, we must use ENMs only when we have sufficient data to estimate the niche under novel conditions. To address this issue, previous studies proposed and tested methods to improve and assess the generality of ENMs. For example, some studies take advantage of the temporal sequence of invasions to compare the performance of ENMs trained only on native occurrences to those trained on occurrences from the native range and early stages of invasion on projecting the environmental suitability for the later stages of invasion (e.g. Barbet‐Massin et al., 2018; Foster et al., 2022). Other studies incorporate occurrence data for closely related species when modelling species' fundamental niches, as a tool to include a broader sampling of environments the species is likely to find suitable and improve model projections (Jiménez & Soberón, 2022). Quantifying the quality of presence data (e.g. spatial resolution) as a pre‐modelling step was also shown to produce more robust ENM projections under out‐of‐sample conditions (Liu et al., 2022). For alien species, we typically have occurrence records in the invasive range, yet we would like to know whether these records are sufficient to characterise the full environmental niche of the species, which is necessary to make accurate range projections. However, we currently lack methods to estimate whether the current knowledge of an alien species distribution is adequate to produce models with the ability to accurately project distributions under unsampled conditions.

Here, we propose a framework, the cumulative niche approach (CNA), for estimating if an alien species' available occurrence records are suitable for describing its environmental niche. The framework is based on using the increase in the realised niche with an accumulated number of occurrences in its native and alien ranges. As we sum the niches a species realises in different regions of its range, we approach the breadth of its fundamental niche (Murray, 2001). If a species has reached environmental equilibrium (in the native or the alien ranges), we expect to see an asymptotic relationship between occurrences and niche breadth. However, if the relationship has not saturated for a species, this would indicate that either its species‐environmental equilibrium has not yet been reached and/or that more occurrence records are needed to estimate it. The CNA allows quantifying whether species have expanded or maintained their environmental niches when colonising new regions and to evaluate whether suitability projections into new areas, or future climatic scenarios, are likely to yield satisfactory results.

## 2 CONCEPTUAL FRAMEWORK

**FIGURE 1:**
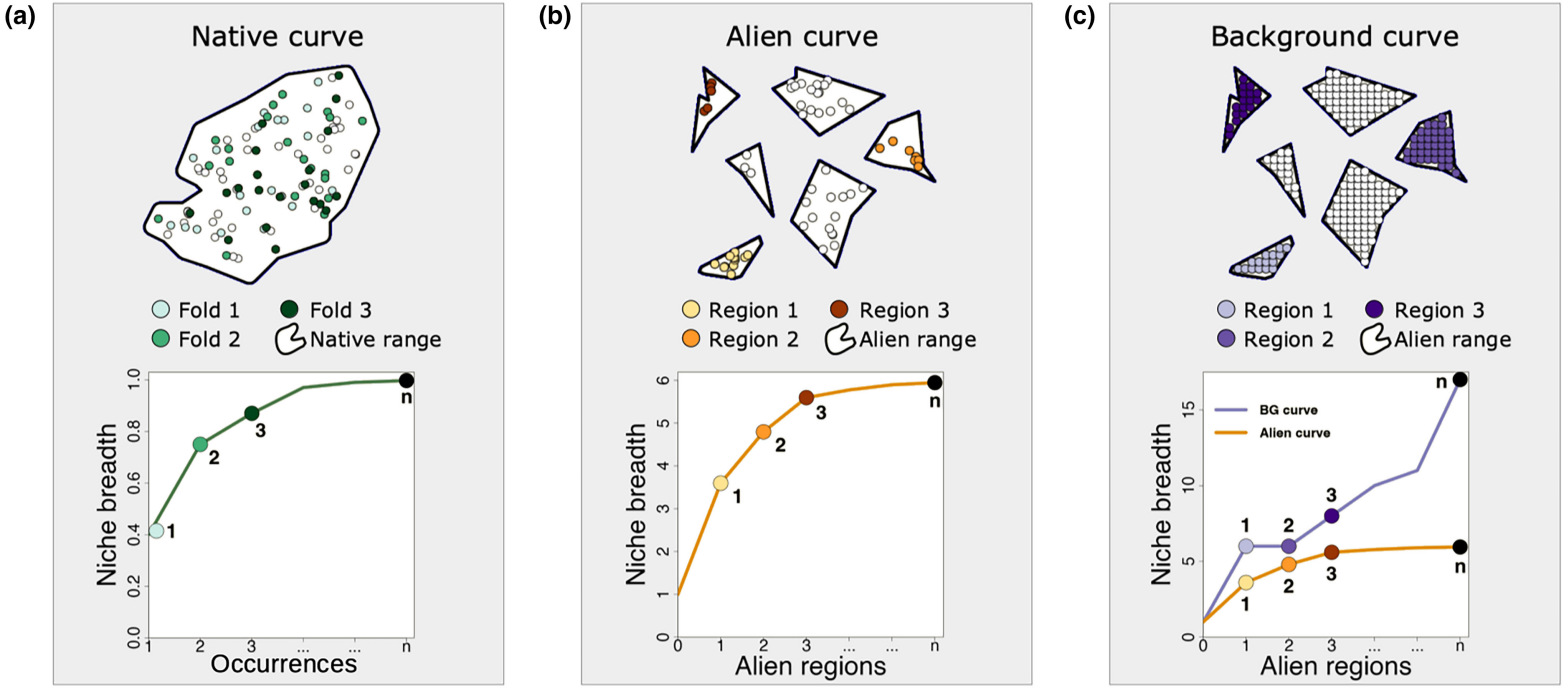
Schemes representing the construction of the accumulation curves. (a) Native distribution occurrence data within the species range map divided in random folds containing equal portions of the occurrence data and the steps of the native curve. (b) Alien occurrence distribution data within each alien checklist region where the species has been reported and the steps of the alien curve. (c) Background points equally distributed within each alien checklist region to represent all conditions available and the alien curve compared to the background curve.

In community ecology, researchers use species accumulation curves to determine whether sampling is sufficient to capture the majority of species present in a defined place, which would be indicated by reaching an asymptote in the relationship between species richness and number of sampling units (Soberón & Llorente, 1993). This linear increase followed by a reaching a plateau for higher numbers of repetitions is also observed in other phenomena following power laws, such as species–area relationship (Dengler, 2009). Similarly, we can expect an asymptotic relationship between the number of occurrences sampled and the niche breadth of a focal species (Murray, 2001), where reaching the asymptote would indicate that our occurrences represent all suitable environments for the species (a species‐environment equilibrium has been reached; Figure 1). Thus, if an asymptotic relationship is observed, and under certain conditions including non‐biased sampling, we can assume that the species' niche inferred from occurrence points is an exhaustive representation of its ecological requirements and ENMs can be used for projections of environmental suitability into new or unsampled conditions.

The CNA framework relies on the comparison of three accumulation curves. First, we produce a native niche accumulation curve, henceforth native curve, ranging from zero to one, where one indicates the full representation of the observed native niche breadth (Figure 1a). All possible native curves will start at zero and end at 1 for the native niche accumulation curve, but the shape of the curve has meaning. If a species shows an asymptotic relationship, this indicates that its native environmental niche is well represented by available occurrence records. Alternatively, if the species has a more linear relationship, gaps in spatial data could have prevented a full representation of the observed native niche breadth (Hortal et al., 2008). These gaps could be due to insufficient reporting, local extinctions or biotic/abiotic barriers preventing the species from occupying all suitable environments in the native range. Second, we produce an alien niche accumulation curve, or alien curve for simplicity, which starts at one, as it includes the niche breadth realised in the native range. As a species is introduced to new regions, it may be exposed to different environmental conditions and expand its realised niche (Andrade et al., 2019; Broennimann et al., 2007). Therefore, it is possible to observe an expansion in the realised niche of a species as it progressively colonises novel conditions. Thus, the alien curve can increase if the species expands its niche breadth as data from alien regions are included (Figure 1b). However, species have intrinsic tolerances to specific conditions, such as temperature (Araújo et al., 2013) and precipitation (Born & Linder, 2018), that define their fundamental niches. When an asymptote is found, we may infer that there is enough knowledge about the species‐environment requirements in the native and in the alien ranges.

Finally, if a species is introduced to regions where the available environmental conditions are not novel (i.e. are similar to the native range), there are no possibilities of niche expansion. That scenario would lead to the alien curve indicating an asymptote for the simple reason that the environmental conditions in the region have a high degree of nestedness within the regions previously included in the model (Parravicini et al., 2015). We suggest controlling for this issue by drawing another curve representing all the conditions available in the new regions, a background accumulation curve (Figure 1c). Only if the background curve indicates that the conditions offered by the regions where a species has been introduced would allow for a greater niche breadth expansion than that realised by the species, would we be able to infer that an asymptote in the alien curve is due to the species' intrinsic limitations.

**FIGURE 2:**
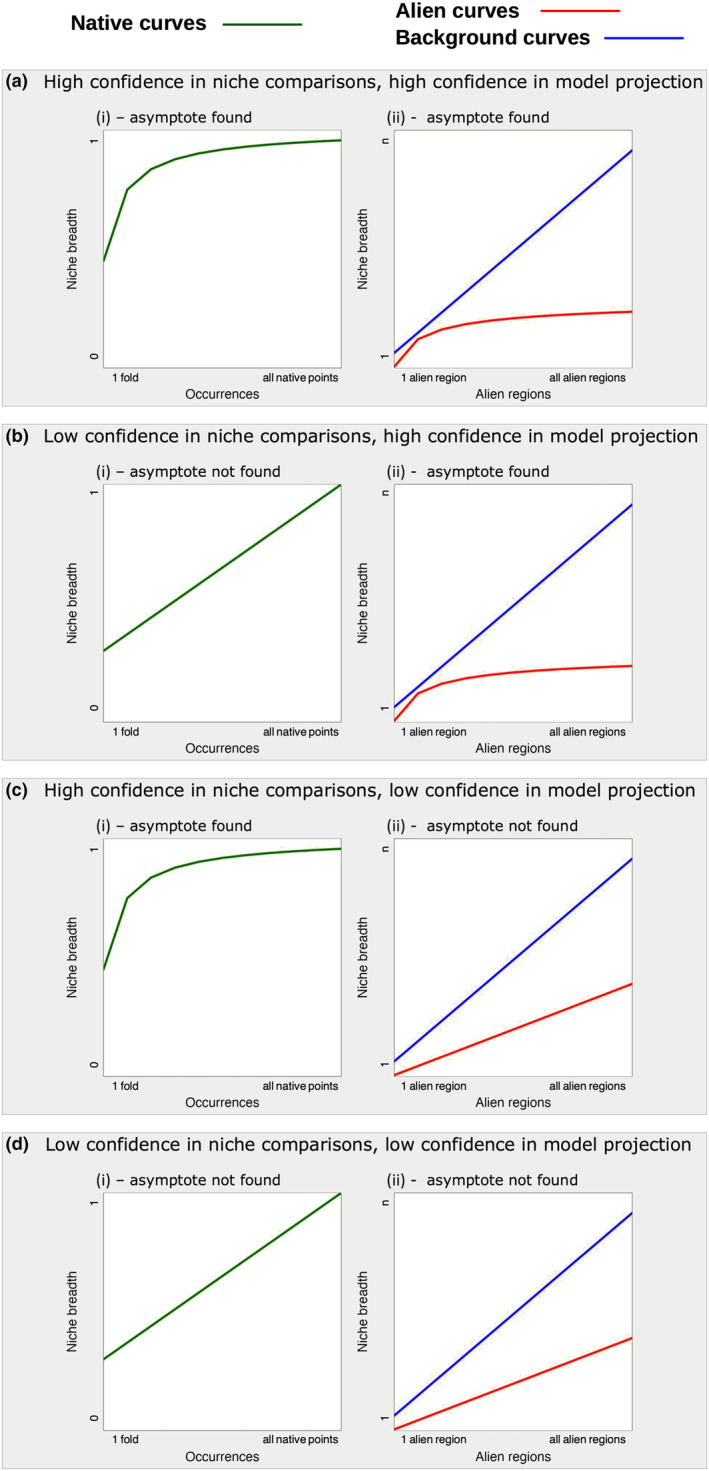
Possible outcome scenarios of the cumulative niche approach. (a) An asymptote is found in both the native and the alien niches of the species distribution. (b) No asymptote is found in the native niche, but the relationship is observed in the alien niche. (c) An asymptote is found in the native realised niche, but not in the alien range. (d) No asymptote is found either in the native or in the alien realised niches. (i) The *x*‐axis shows the number of folds containing the records used to calculate the realised niche in the native range, and the *y*‐axis shows the proportion of the total realised native niche calculated with the partial data. The green line shows the native curve. (ii) The *x*‐axis shows the number of alien regions contributing to the niche breadth calculation, and the *y*‐axis shows the increase in the realised niche, using the native realised niche as the base for the calculation. The red line represents the alien curve, and the blue line represents the background curve.

Taken together, the native and alien curves allow us to assess the adequacy of species' occurrence data to answer questions about niche expansions. Provided an asymptote is found in the native curve, an increase in the total niche breadth in the alien curve represents a real niche expansion between the native and the alien ranges (Figure 2a,c). This is done by comparing the niche a species realises in its native range to that realised in the regions where it has been introduced (Bates & Bertelsmeier, 2021). If an asymptote is reached for the native curve, we can assert that there is enough knowledge to characterise the species' native realised niche (Figure 2a–i, ci). This would give us high confidence in niche comparisons. If an asymptote is not found in the native curve (Figure 2bi,di), it shows we have uncertainty in the species' realised native niche breadth and a poor ability to answer questions about niche expansions (Fründ et al., 2016). Such results are due to either gaps in the data or to species being pushed out of equilibrium in their native range by anthropogenic pressures that caused range contraction or expansion (Faurby & Araújo, 2018).

The second result, represented by the alien and the background curves, indicates whether available data on the species distribution are sufficient for sound projections into new or unsampled conditions. Reaching an asymptote in the alien curve (Figure 2aii,bii) indicates that there is enough knowledge to characterise the species' realised niche, implying high confidence in model projections. This can only be meaningful if the conditions offered by the regions where the species is found (represented by the background curve) allow for a greater niche expansion than the one observed. The background curve will reach an asymptote if the conditions in the new regions added to the model are nested within those previously included. In these cases, the alien curve will naturally appear flat; however, this is due to environmental limitations, instead of those intrinsic to the species (e.g. Araújo et al., 2013; Born & Linder, 2018). Thus, finding an asymptote in the alien curve, coupled with a greater expansion in the background curve, indicates that the species distribution data are adequate to produce an ENM suitable for projections into new or unsampled conditions. When the possibilities of niche expansion (background curve) continue to increase and the alien curve does not reach an asymptote (Figure 2cii,dii), we may conclude that there is not enough data to infer if the species is in equilibrium with the environment. ENM projections into new or unsampled conditions based on these data will most likely have low predictive power. Nevertheless, this context indicates that the species is increasing its realised niche as it is exposed to new environmental conditions.

## 3 EXAMPLE

To illustrate the CNA conceptual framework, we selected 26 species of amphibian, bird and mammal species that have established outside their native ranges as study cases. We selected the species based on their broad alien distribution (introduced to at least 10 regions of the world) and the availability of range maps of their native ranges. The scripts used for all steps described subsequently are available at https://github.com/EduardoArle/CNA.

### 3.1 Data acquisition and preparation

**FIGURE 3:**
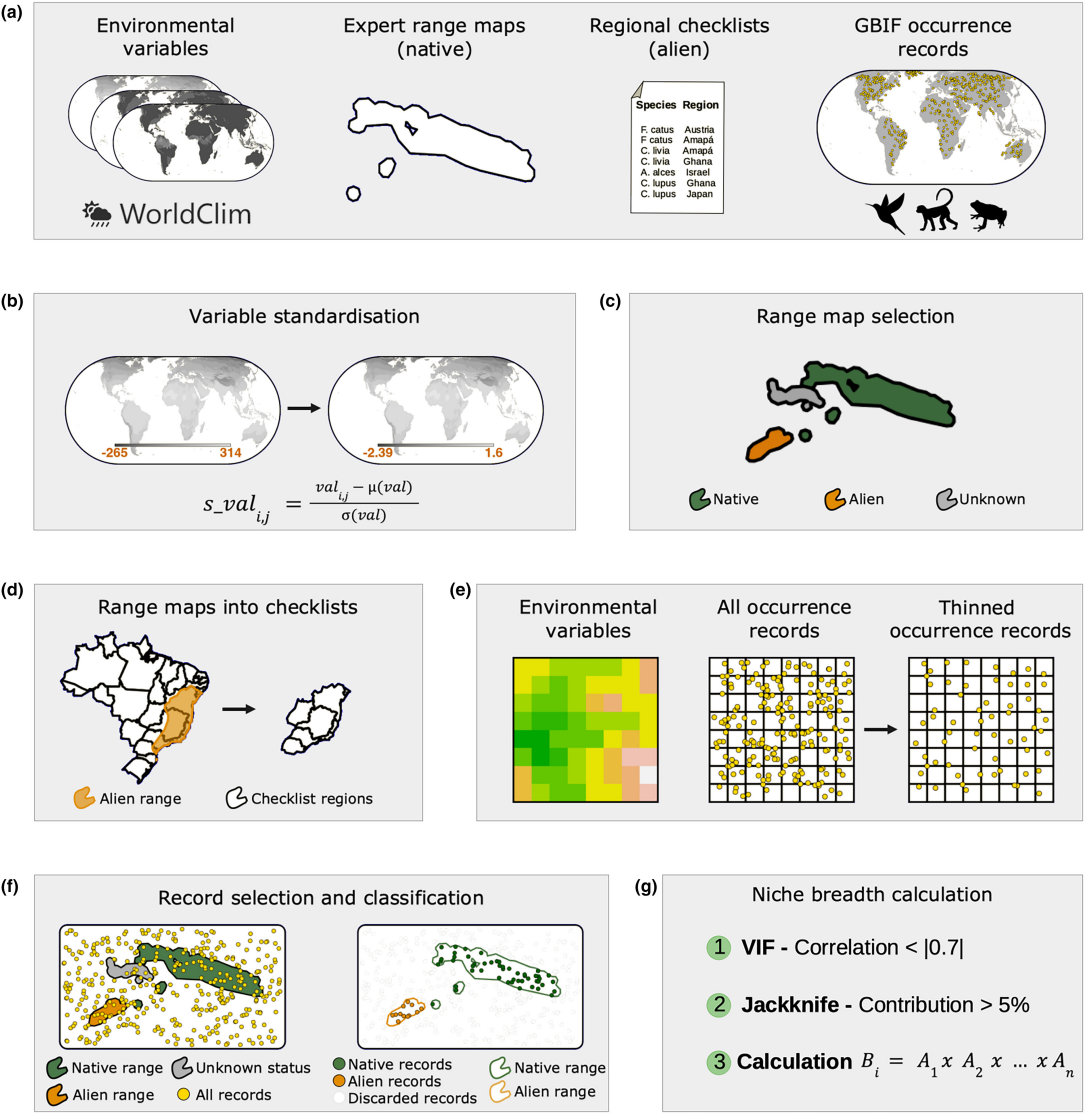
Steps in data acquisition and preparation to develop the showcases of the CNA framework (a–g): (a) Required data. (b) Variable standardisation to calculate the species' niche breadth. (c) Selection of the features representing the species' native range. (d) Transformation of alien species range maps of the DAMA database (Biancolini et al., 2021) into checklists providing high geographical completeness, comprising all landmasses divided into administrative units and islands. (e) Thinning of occurrence records according to the environmental variables' spatial resolution. (f) Selection and biogeographical classification of records falling within the species' known native and alien distribution. (g) Steps to select variables and calculate the species' niche breadth.

Four types of data were required: (1) environmental variables; (2) expert‐based range maps for the species' native distributions; (3) checklists indicating the regions where the species have been introduced; and (4) species occurrence records, that is coordinates of species presence (Figure 3a).

We obtained 19 bioclimatic variables from the widely used WorldClim 2.1. database (WorldClim, 2021) at a 2.5 arc‐minute resolution using the function *getData* in the R‐package ‘raster’ (Hijmans & van Etten, 2012). To calculate the species' niche breadth, we produced standardised values (Cardillo et al., 2019) for each variable ($s_{\mathrm{va}}l_{i,j}$) by

(1) $$s_{\mathrm{va}}l_{i,j}=\frac{\mathrm{va}l_{i,j}-\mu \;\left(\mathrm{val}\right)}{\sigma \;\left(\mathrm{val}\right)},$$

where $\mathrm{va}l_{i,j}$ represents the original values in each variable raster layer, $\mu \;\left(\mathrm{val}\right)$ and $\sigma \;\left(\mathrm{val}\right)$ are the average and the standard deviation of each layer (Figure 3b).

We used species range maps to describe the boundaries in which a species is expected to be found natively (Jetz et al., 2012). We obtained range maps representing the species' native distributions from the IUCN's and BirdLife's Red Lists of Threatened Species (Birds of the World, 2022; IUCN, 2021). These maps were processed to remove areas in which a species' biogeographical status was ‘Introduced’ or ‘Unknown’ (Figure 3c).

We inferred the species' alien distributions from administrative area checklists for amphibians and birds (Capinha et al., 2017; Dyer et al., 2017), and expert‐based range maps for mammals (Biancolini et al., 2021). To showcase the CNA framework, we represented alien distributions of all species only as combinations of occupied administrative units, due to the higher availability of checklist data compared to expert range maps for alien ranges. Therefore, we transformed mammal alien range data into checklists by overlaying the original range maps with an administrative areas shapefile (Guénard et al., 2017; Figure 3d). The shapefiles containing the regions used in this study are freely available online (see Section 4: data availability statement).

We obtained the occurrence records for all species from the Global Biodiversity Information Facility (GBIF.org, 2022). We thinned the occurrence data for each species by keeping only one record per 2.5 arc‐minute cell, the environmental variables' resolution (Figure 3e). We discarded any records not overlapping either the native or the alien regions (Figure 3f).

### 3.2 Variable selection and niche breadth calculation

For the purpose of calculating the species' niche breadths, we selected environmental layers for each species amongst the 19 WorldClim variables. The variable selection process consisted of two steps: (1) identifying variables with lower pairwise correlation and (2) selecting those with higher explanatory power to model the species' distribution. We calculated the variance inflation factor (VIF), selecting only variables with a correlation coefficient smaller than |0.7| (R‐package ‘usdm’ – Naimi, 2015). This procedure aims to reduce problems caused by collinearity between explanatory variables (Naimi & Araújo, 2016). Subsequently, we fitted an EMN with the variables selected in the previous step as environmental predictors using the Maxent algorithm in the R‐package ‘dismo’ (Hijmans et al., 2017). The ENMs were fitted with the default settings aiming to calculate the explanatory power of each variable to the species distribution. Finally, we selected the variables that contributed at least 5% to projecting the species distribution using the jackknife variable selection criterion (Figure 3g; Cobos et al., 2019; Nath & Jones, 1988).

We calculate the total breadth ($B_{i}$) of the realised native niche of each species in a simple approach to showcase our framework:

(2) $$B_{i}=A_{1}xA_{2}x\ldots xA_{n},$$

where $A_{j}$ is the amplitude of each selected variable (using the standardised version; Figure 3g). This multiplication of the ranges allows us to estimate the multidimensional volume of the environmental space occupied by a species. The resulting value is the basis for the subsequent analyses, namely the native and the alien niche accumulation curves.

### 3.3 Native niche accumulation curves

We calculate the total native niche breadth of each species considering all native occurrences, this value is the reference for all subsequent niche breadth comparisons. The native curve calculation consisted of partitioning the native occurrence data into several folds and progressively calculating the proportion of the environmental space represented by the partial data. The number of folds and the amount of data points within each fold depended on the total number of occurrences available for each species. For species with more than 1000 occurrences, we created *n* folds of 100 occurrences; species with less than 1000 and more than 30 occurrences were partitioned into folds containing 10 occurrences; and for those species represented by 30 records or less, each fold had one single occurrence. We then calculated the native curve in *n* iterations, where *n* is the number of folds created for the species occurrences. In each iteration, a new fold is added to the data, and the resulting niche breadth is divided by the reference niche breadth value, resulting in a partial niche breadth value between zero and one. This cumulative process results in higher values after each iteration, being always one at the *n*th iteration (Figure 1a). We repeated the process 10 times and considered the average values to produce the native curve.

### 3.4 Alien niche accumulation curves and background curves

The niche accumulation in the alien range of each species is represented by two curves, the background showing the maximum niche expansion offered by the conditions available in the regions, and the alien curve showing the actual niche expansion realised by the species (Figure 1c).

We calculated the alien curves in *n* iterations, *n* being the number of regions where a species has been listed as introduced. In the first iteration, the occurrences located in the firstly selected alien region are combined to the native occurrences, and the resulting niche breadth is divided by the total native niche breadth of the species, resulting in a value greater than or equal to one. In each subsequent iteration, occurrences corresponding to a new region are added to the data. The resulting values may increase as more data accumulates after each iteration, indicating an expansion of the realised niche of the species (Figure 1b). To control for the order of inclusion of each region, we repeated the process 10 times, considering the average values to calculate the alien curve.

For the calculation of the background curve, we randomly selected one point per 0.25° grid cell (corresponding to ~25 × 25 km at the equator) in each of the regions selected during the construction of the alien curve and extracted the values of all selected variables in those localities.

## 4 RESULTS AND PATTERNS DESCRIPTION

The 26 species we used to showcase the CNA concept produced different accumulation curves both in the native and alien range analyses. Here, we present four cases to demonstrate different outcomes of our framework and further discuss the implications of the CNA results for these species. Graphs showing the results for all species are available in Figure S1.

**FIGURE 4:**
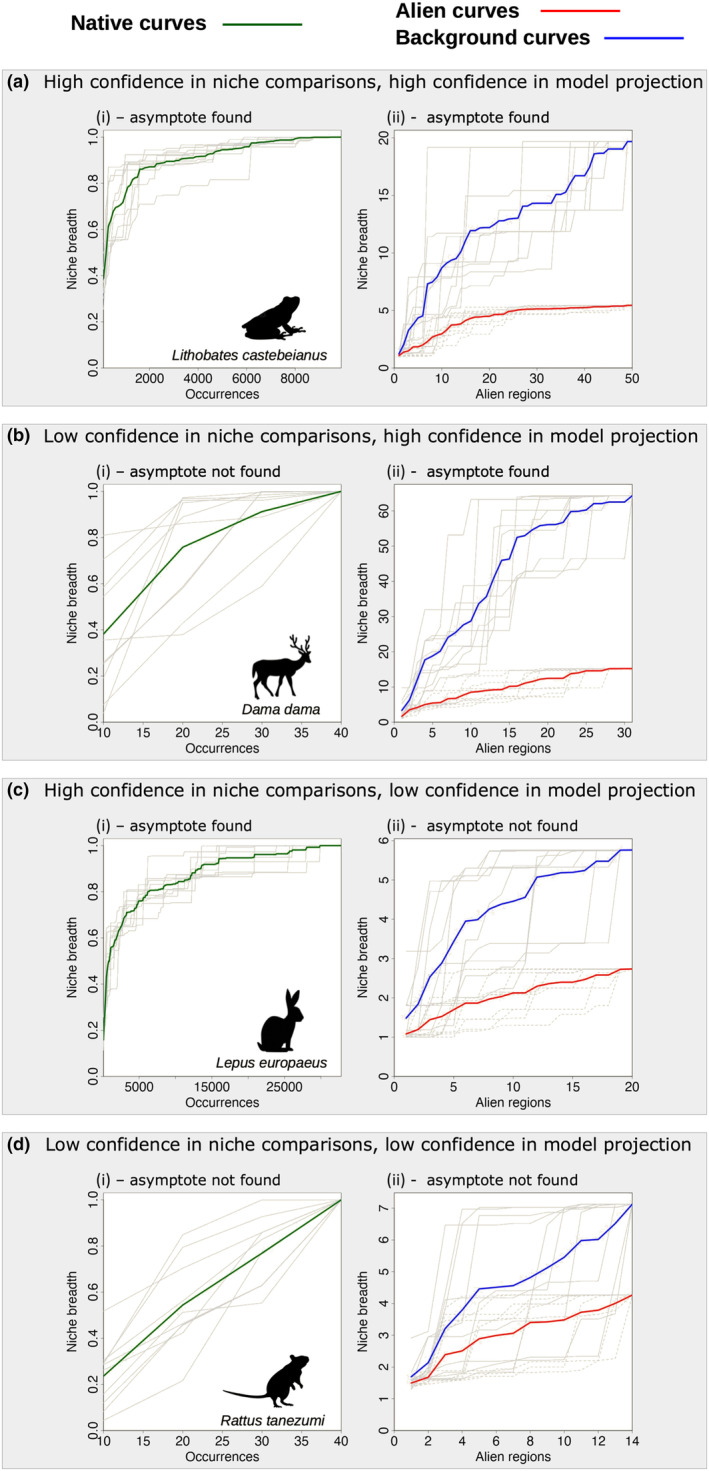
Examples of the niche accumulation curves representing the four CNA possible outcomes for the species: (a) *Lithobates castebeianus*; (b) *Dama dama*; (c) *Lepus europaeus*; and (d) *Rattus tanezumi*. (i) native curve for each species. The *x*‐axis shows the number of records used to calculate the realised niche in the native range, and the *y*‐axis shows the proportion of the total realised native niche calculated with the partial data. Grey lines represent each of the repetitions, and the green line is the average of the 10 repetitions. (ii) alien curve for each species. The *x*‐axis shows the number of alien regions contributing to the niche breadth calculation, and the *y*‐axis shows the increase in the realised niche, using the native realised niche as the base for the calculation. Dashed lines in grey represent each repetition of the alien curve, and the red line is the average of the 10 repetitions. Full grey lines represent the background curve, and the blue line is the average amongst the repetitions.

The American bullfrog (*Lithobates castebeianus*) exemplifies the scenario of high confidence in niche comparisons and high confidence in model projections. The species is well represented by occurrence records in its native range: as the native curve approaches an asymptote, with less than 50% of the data being sufficient to calculate more than 90% of its native niche breadth (Figure 4ai). In the alien range, the species showed a niche expansion of approximately five times the size (in terms of covered environmental space) of the niche realised in the native range. As the regions where the species has established alien populations are progressively added to the model, the potential conditions for niche expansion, represented by the background curve, increases continuously. However, the alien curve of the species approaches an asymptote with less than 50% of the regions being included in the model (Figure 4aii). This clear asymptote signals that the realised niche of the American bullfrog is well represented by the available occurrence points and that this species will probably not further increase its realised niche when new occurrence points become available. We suggest that the patterns found here indicate that ENMs for this species will likely allow for robust projections of environmental suitability into new areas not yet occupied by the American bullfrog.

The European fallow deer *(Dama dama*) shows low confidence in niche comparisons and high confidence in model projections. The native curve does not reach an asymptote, as more observations are added to the model, the niche breadth increases continuously (Figure 4bi). Thus, the increase in the realised niche observed when producing the alien curve does not necessarily represent an actual niche expansion. This apparent expansion of the realised niche likely reflects, at least partly, that the actually realised niche in the native range is not comprehensively represented by the available occurrence records. Conversely, the *D. dama* distribution is well represented in several of the regions where it is alien, producing an alien curve that approaches an asymptote. Moreover, the conditions offered by the alien regions increase continuously, as it is shown in the background curve (Figure 4bii). This example shows a case in which no assumptions about niche expansion between native and alien ranges can be made. However, robust projections of environmental suitability into new areas not yet occupied should be possible.

The European hare (*Lepus europaeus*) represents a case of high confidence in niche comparisons and low confidence in model projections. There is enough data to reach an asymptote in the native curve (Figure 4ci), and the alien curve shows that niche expansion occurs, but an asymptote is not found. Although the species does not exploit all the conditions offered by the new regions, the alien curve is still ascending when it reaches circa three times the size of the native niche breadth, indicating that the alien niche breadth keeps increasing as new introductions take place (Figure 4cii). These patterns do not point to a species‐environmental equilibrium, suggesting that the realised niche of *L. europaeus* is likely to expand when new invasions (new occurrence points) occur. We suggest that ENMs projections of environmental suitability in currently unoccupied areas would not be reliable.

Finally, the Asian rat (*Rattus tanezumi*) showcases the scenario of low confidence in niche comparisons and low confidence in model projections. The species is not well represented by occurrence records in its native range, so its native curve does not reach an asymptote (Figure 4di). Likewise, the alien curve does not reach an asymptote (Figure 4dii). Thus, it is impossible to deduce niche expansion or to make robust projections into new areas not yet occupied.

## 5 DISCUSSION

The CNA framework can be used to assess the data adequacy to measure species' realised niches in their native and alien ranges and to quantify niche expansion in biological invasion scenarios. In addition, our CNA results can evaluate whether our knowledge of species' accumulated realised niches across different portions of the geographical distribution is sufficient to produce ENMs that would be expected to reasonably assess the environmental suitability of new areas the species has not yet invaded. The limitations of applying ENMs to invasion biology are comparable to those found in studies aiming to model species' future distribution, as both applications rely on model transferability (Heikkinen et al., 2012; Qiao et al., 2019). Thus, the CNA can be used as a tool to reduce the uncertainties inherent to model projections into new regions or under climate change scenarios.

The four alien species chosen to demonstrate the possible outcomes of the framework are alien to several regions of the world where they are known to cause serious ecological and economic impacts. The American bullfrog, native to Eastern North America, has been introduced in more than 40 countries for commercial purposes over the last century, causing serious damage to the local amphibian populations, especially due to the large size of tadpoles leading to predation on native amphibian juveniles (Ficetola et al., 2007). The European fallow deer has a long history of introductions, dating back to Neolithic times (Sykes, 2004) and continuing until recently, often as game species, posing risks to the local environment, for example, as a vector for foot‐and‐mouth disease (Pereira‐Garbero et al., 2013). Moreover, the original range of this deer species is poorly known due to the long human induced modifications in its native habitat (Karastoyanova et al., 2020). Finding an asymptote for these two species using occurrences from the native and alien ranges indicates the possibility of producing robust risk assessments and guide efforts to prevent further spread, such as the recent prediction of the European fallow deer range expansion in Tasmania (Cunningham et al., 2022). The European hare is known to spread rapidly when introduced to suitable new areas, often out‐competing native hare species (Caravaggi et al., 2015) and becoming a crop pest (Bonino et al., 2010). Despite having been introduced to more than 20 new regions, *Lepus europaeus* continues to increase its total realised niche breadth, as indicated by the alien curve not reaching an asymptote. The same pattern is found in the Asian rat's niche expansion, a hazard to human health as a vector of diseases (Morand et al., 2015) and a threat to native biodiversity, competing with native rodents and predating on birds and eggs (Harper & Bunbury, 2015). These two species keep increasing their realised niche as new introductions occur, and more occurrence data are needed to improve the knowledge on the species' niches.

The methods adopted in this study aim to illustrate the concept of the CNA, and future studies can flexibly modify this approach. For example, it is possible to use alternative methods to calculate niche size (e.g. Dolédec et al., 2000; Feinsinger et al., 1981; Fridley et al., 2007; Warren et al., 2008). The environmental variables we used were all bioclimatic (precipitation and temperature). Future studies could consider other variables that might be particularly relevant to the focal species, such as soil characteristics (Gardner et al., 2019), slope (Nezer et al., 2017), salinity and depth in the case of marine studies (Bentlage et al., 2013) and biotic interactions (Gherghel et al., 2018). Including those variables in the models is challenging, but may provide a more meaningful description of the environmental niche. Another technical aspect that can be changed is how the alien regions are defined. We relied on administrative area checklists, which may differ greatly depending on the country and are frequently large. As a result, the background curve can be overestimated in larger regions and underestimated in smaller ones. Furthermore, their boundaries are political rather than ecological, resulting in the exclusion or inclusion of areas that would be within or outside of the reach of species. Alternative distribution data sources, such as alien range maps, may be available. If no explicit information on the alien distribution of a species exists, there are methods to estimate the biogeographical status of individual occurrence points based on the known native distribution (e.g. Arlé et al., 2021). Point occurrence data can be used to generate convex hulls (Meyer et al., 2017) or gridded data (Staude et al., 2020) to represent the invaded regions. Integrating intraspecific variation in ENMs has been proposed in recent studies (e.g. Chardon et al., 2020; Zhang & Kubota, 2021) and can be a promising adaptation of the CNA framework, provided the adequate genetic data is available. Finally, knowing the invasion history of the focal species can guide the order of inclusion of the regions (e.g. Seebens, 2016) and provide more insights about the niche expansion.

To further develop the CNA into a systematic methodology, the results could be compared against the results of mechanistic models (Kearney & Porter, 2009). These tests are often infeasible for many species due to limitations in data availability and the need of specific knowledge about the mechanisms determining the species fundamental niche, such as physiological tolerances to abiotic factors and species interactions (Alvarado‐Serrano & Knowles, 2014; Peterson et al., 2015; Spence & Tingley, 2020). However, comparative tests considering species for which the necessary data for mechanistic models is available could validate the concept and generality in the applications of the CNA framework (e.g. Fordham et al., 2018; Ponti et al., 2021).

The presented framework assumes that occurrence records used for analysing species' native and alien niches are unbiased with respect to relevant environmental gradients. Under biased sampling, it is possible that detected asymptotic curves might instead only capture the well‐sampled portions of species' realised niches. Given the prevalence of sampling biases in many datasets (Meyer et al., 2016), modellers should screen data for possible biases before applying the standard CNA framework and take appropriate measures to control for them (e.g. via environmental filtering; Varela et al., 2014). For example, general sampling biases may be detected by comparing accumulation curves across all available species records against curves for stratified‐randomly sampled locations, whilst species‐specific biases may be ruled out by comparing curves for focal species against curves for larger species groups that would have plausibly been collected via similar sampling processes. In the latter case, finding a focal species' curve's asymptote around a substantially narrower environmental space than that covered by all records of the reference group should indicate that collectors who would have likely recorded the focal species had sampled a broader environmental space without detecting it there, thus increasing our confidence that the focal species' asymptotic curve captures its true realised environmental niche.

Another caveat is that the realised niche of alien species may be greater than that which would be estimated based on its distribution in the native range (e.g. Andrade et al., 2019; Broennimann et al., 2007). Hence, environmental data may not be sufficient to describe its alien range. The mechanisms underlying this phenomenon are numerous (Goldberg & Lande, 2007; Sexton et al., 2009), ranging from geographical barriers to dispersal (Pulliam, 2000) through altered biotic interactions (e.g. Guisan et al., 2014; Tingley et al., 2014), to rapid evolution under novel environments (e.g. Gering et al., 2019; van Kleunen et al., 2018). However, the aforementioned limitations are shared by all correlative ENM approaches.

## 6 CONCLUSIONS

We developed a framework to evaluate the level of confidence in niche comparisons and in ENM projections into unsampled conditions. As a theoretical framework, the CNA does not aim to consider all the particularities inherent to ecological modelling. Instead, our work can serve as a basis for the development of methods and workflows to improve how we analyse and interpret results aimed at quantifying niche expansion in the alien range and projecting which habitats might be suitable for future invasions. With this manuscript, we provide guidelines to assess the potentiality of model projections based on species' distribution data, supporting more robust forecasting of biological invasions and species distribution under global change scenarios.

## AUTHOR CONTRIBUTIONS

**Eduardo Arlé:** Conceptualization (equal); data curation (lead); formal analysis (lead); funding acquisition (equal); investigation (lead); methodology (lead); project administration (lead); resources (equal); software (lead); validation (equal); visualization (lead); writing – original draft (lead); writing – review and editing (equal). **Tiffany Marie Knight:** Conceptualization (equal); funding acquisition (equal); investigation (supporting); methodology (supporting); project administration (supporting); resources (equal); supervision (supporting); validation (equal); visualization (supporting); writing – original draft (supporting); writing – review and editing (equal). **Marina Jiménez‐Muñoz:** Formal analysis (supporting); investigation (supporting); methodology (supporting); software (supporting); validation (equal); visualization (supporting); writing – review and editing (equal). **Dino Biancolini:** Investigation (supporting); methodology (supporting); validation (equal); visualization (supporting); writing – review and editing (equal). **Jonathan Belmaker:** Conceptualization (equal); formal analysis (supporting); funding acquisition (equal); investigation (supporting); methodology (supporting); supervision (supporting); validation (equal); visualization (supporting); writing – original draft (supporting); writing – review and editing (equal). **Carsten Meyer:** Conceptualization (equal); data curation (supporting); formal analysis (supporting); funding acquisition (equal); investigation (supporting); methodology (supporting); project administration (supporting); resources (equal); software (supporting); supervision (lead); validation (equal); visualization (supporting); writing – original draft (supporting); writing – review and editing (equal).

## CONFLICT OF INTEREST STATEMENT

The authors declare no conflict of interest.

### OPEN RESEARCH BADGES

This article has earned Open Data and Open Materials badges. Data and materials are available at [https://zenodo.org/records/6457868 (DOI: https://doi.org/10.5281/zenodo.6457868); https://zenodo.org/records/6457783 (DOI: https://doi.org/10.5281/zenodo.6457783); https://github.com/EduardoArle/CNA].

## Supporting information


Figure S1.

## Data Availability

All data used in this work is available on Zenodo, DOI: https://doi.org/10.5281/zenodo.6457868; https://doi.org/10.5281/zenodo.6457783 and all code used is available on GitHub, https://github.com/EduardoArle/CNA.
