# Supplementary material for: The cumulative niche approach: A framework to assess the performance of ecological niche model projections
Source: Ecol Evol. 2024 Feb 21;14(2):e11060. doi: 10.1002/ece3.11060 (PMC10880136; doi:10.1002/ece3.11060)

Supplementary Information

**The Cumulative Niche Approach: a framework to assess the performance of ecological niche model projections**

**Fig. S1** – Niche accumulation curves for the 26 species analysed in this work. **i-** Native niche accumulation curves. The x-axis shows the number of records used to calculate the realised niche in the native range, and the y-axis shows the proportion of the total realised native niche calculated with the partial data. Curves in grey represent each of the repetitions, and the thicker green curve is the average of the ten repetitions. **ii-** Alien niche accumulation curve. The x-axis shows the number of alien regions contributing to the niche breadth calculation, and the y-axis shows the increase in the realised niche, using the native realised niche as the base for the calculation. Full lines in grey represent each repetition of the niche accumulation curve, and the red line is the average of the ten repetitions. Dashed grey lines represent the accumulation of conditions offered by the regions included in the analysis, and the blue line is the average amongst the repetitions.

**A** - *Eleutherodactylus johnstonei* (Amphibia)


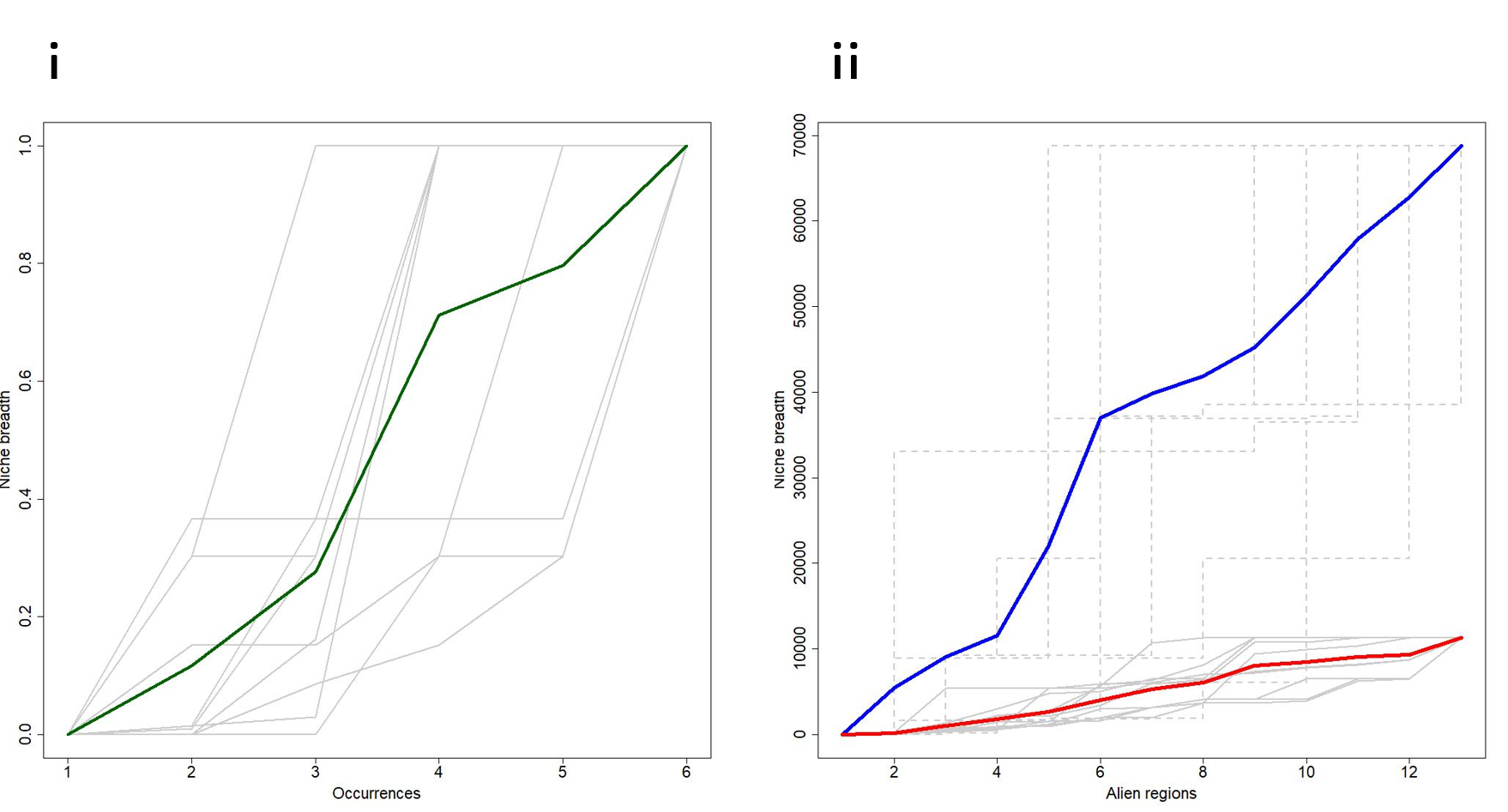


**B** - *Eleutherodactylus planirostris* (Amphibia)


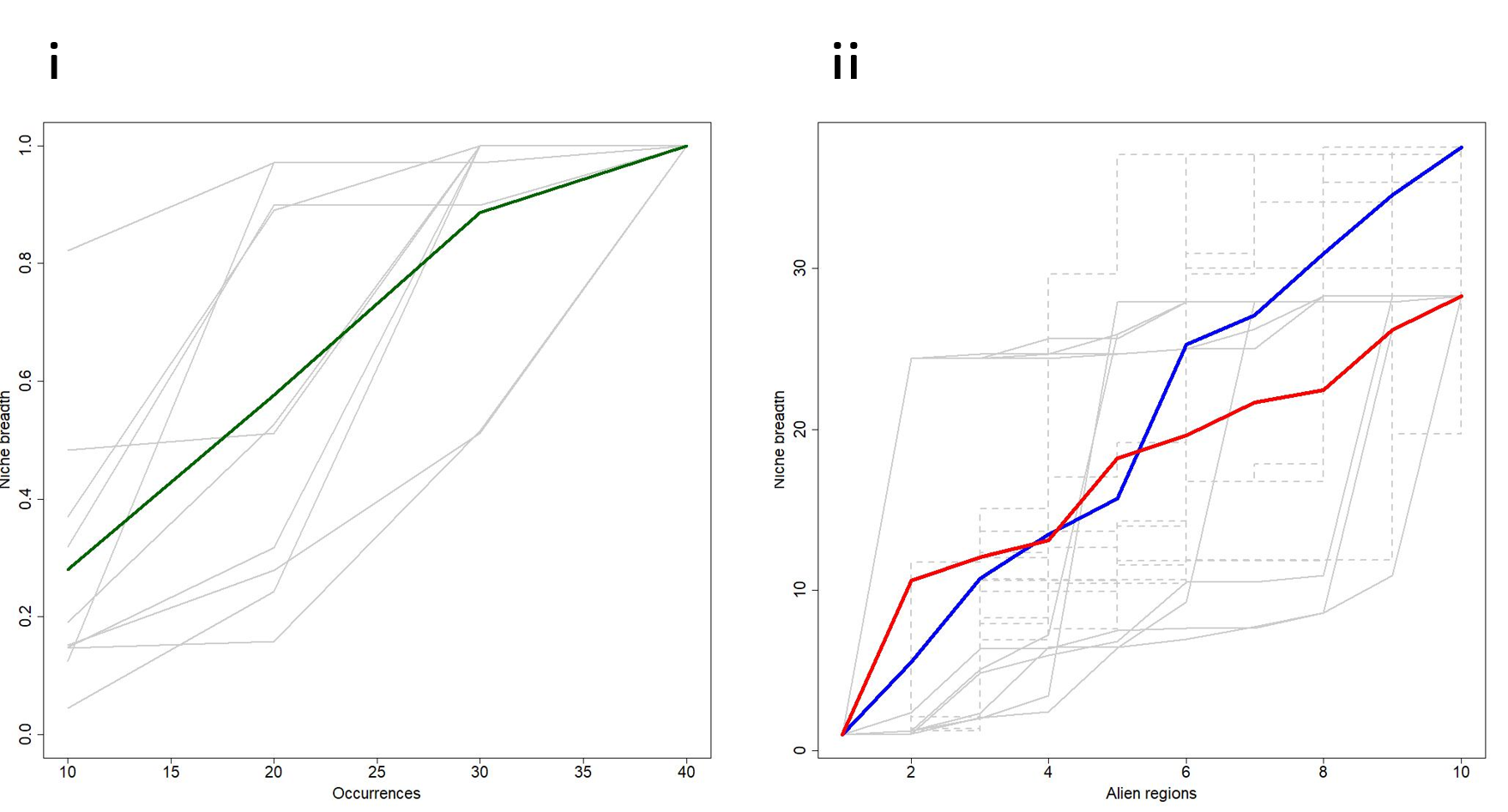


**C** - *Lithobates castebeianus* (Amphibia)


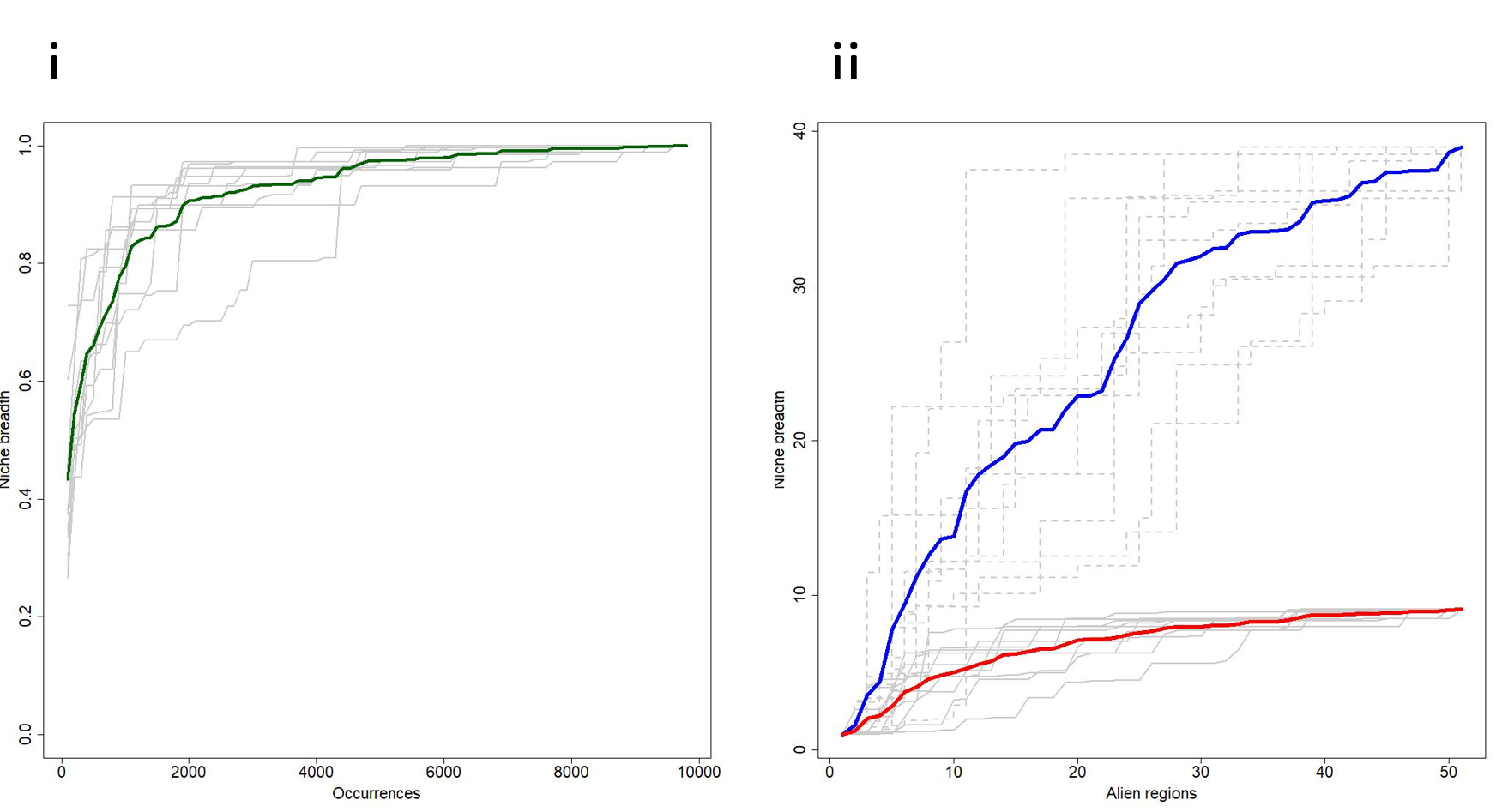


**D** - *Apodemus sylvaticus* (Mammalia)


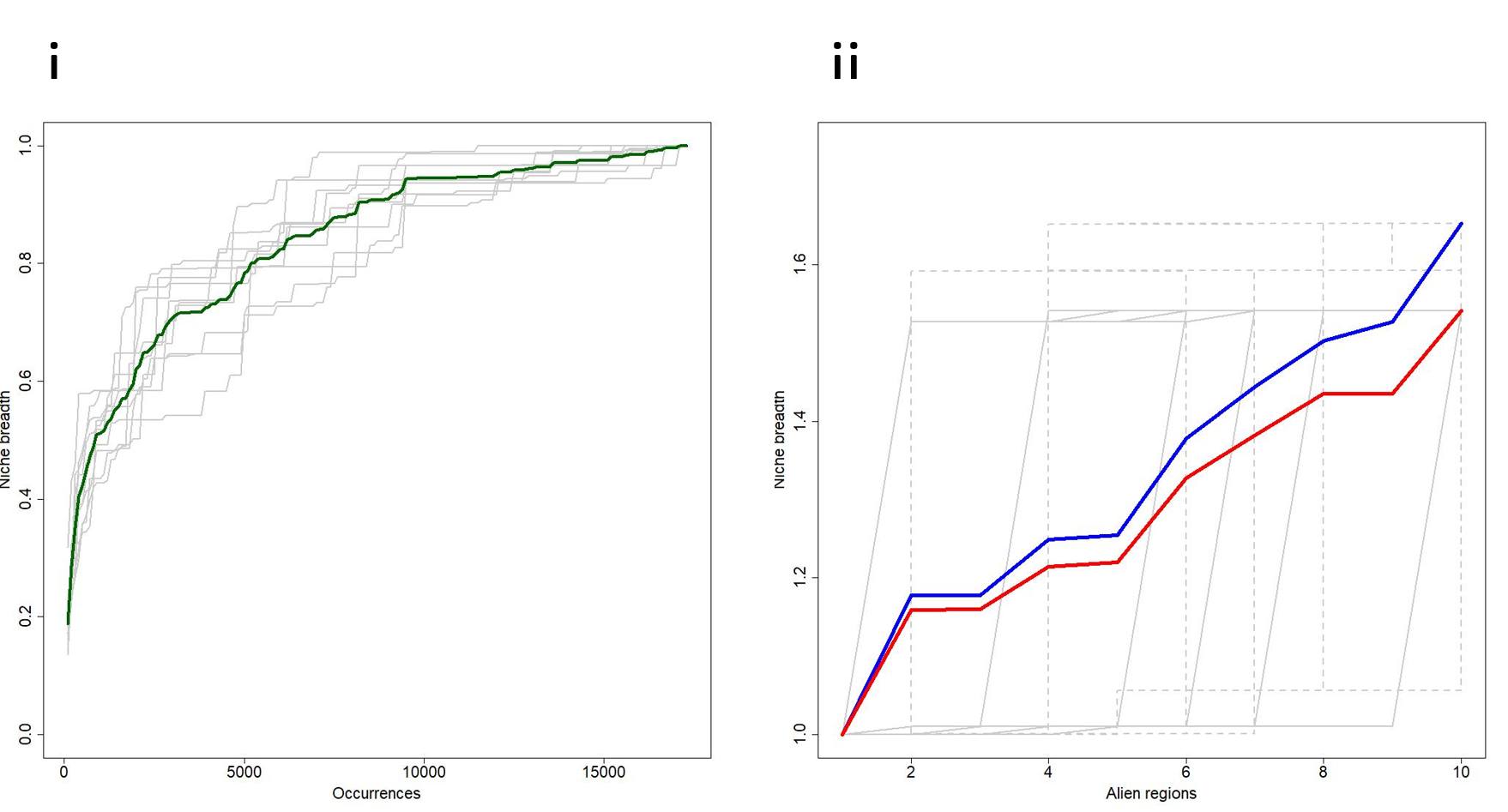


**E** - *Cervus elaphus* (Mammalia)


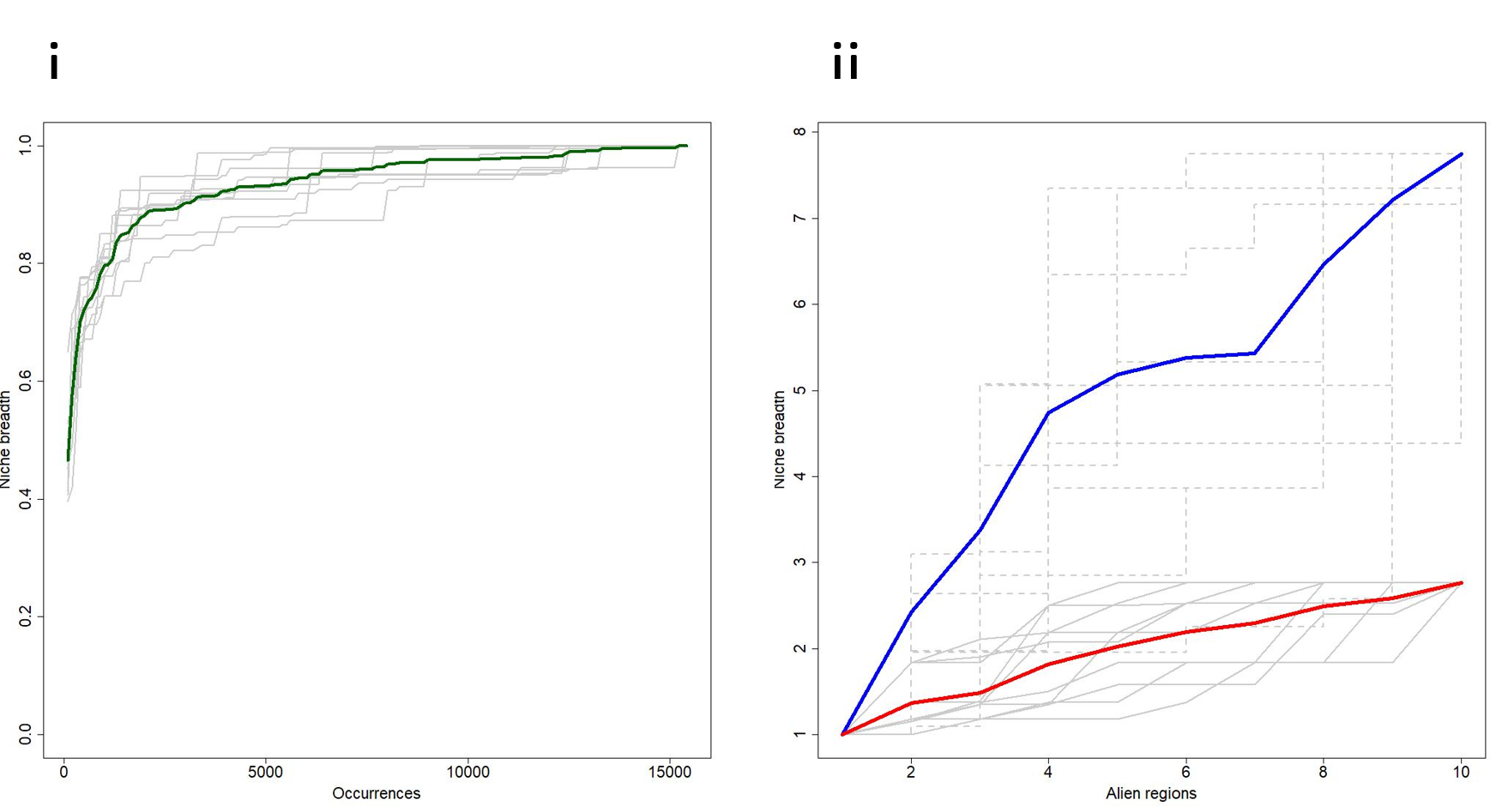


**F** - *Cervus nippon* (Mammalia)


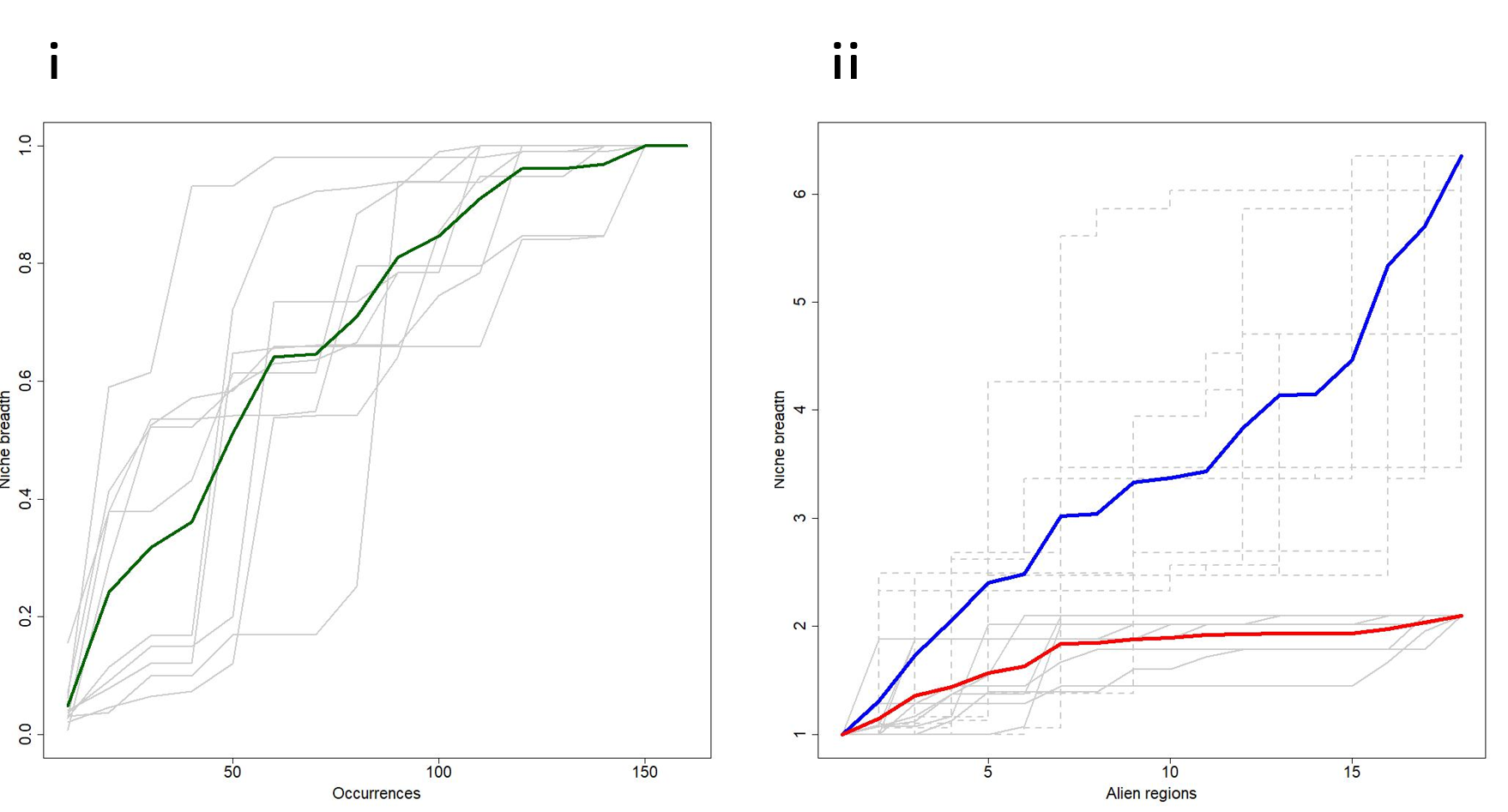


**G** - *Dama dama* (Mammalia)


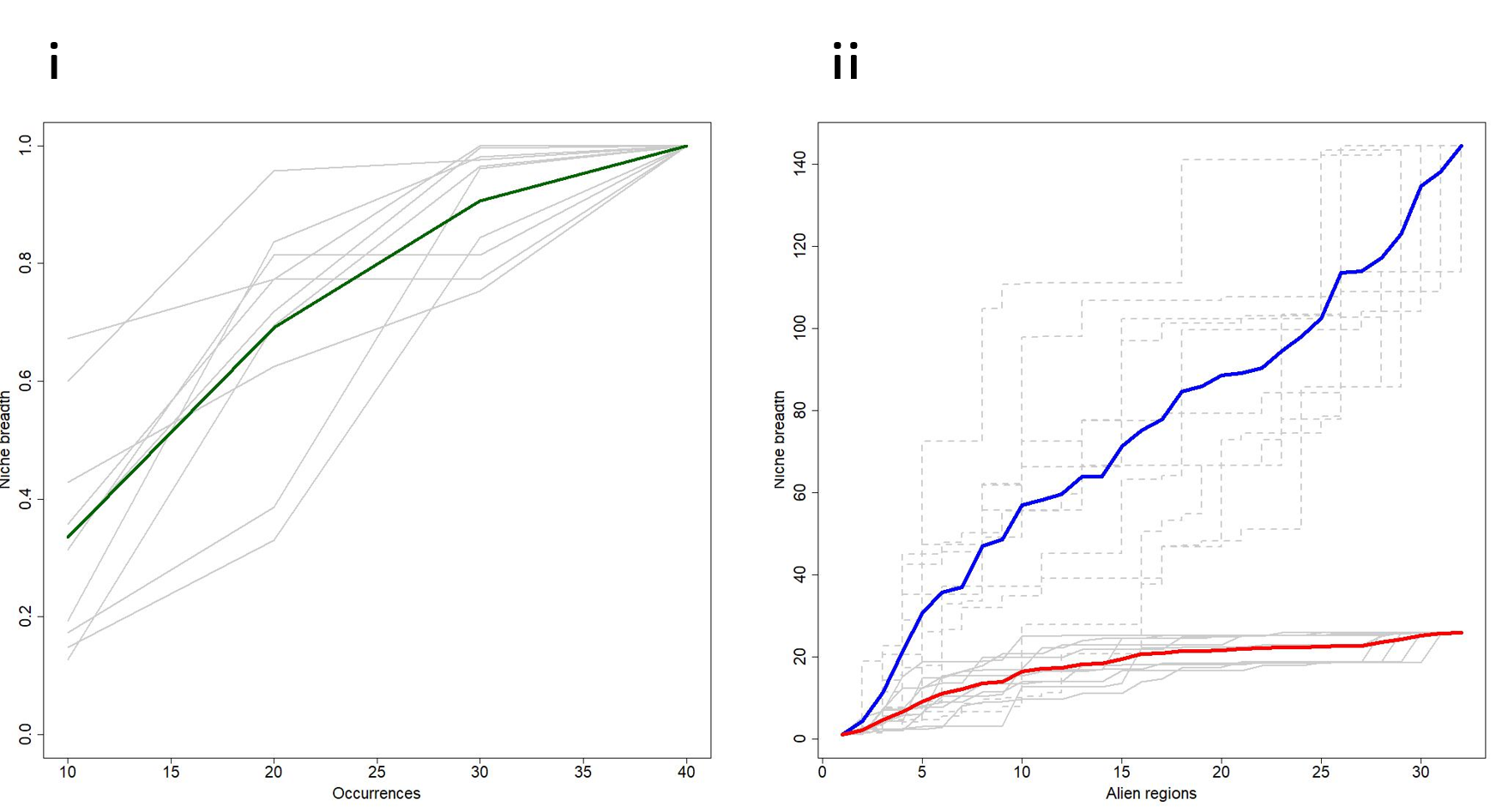


**H** - *Erinaceus europaeus* (Mammalia)


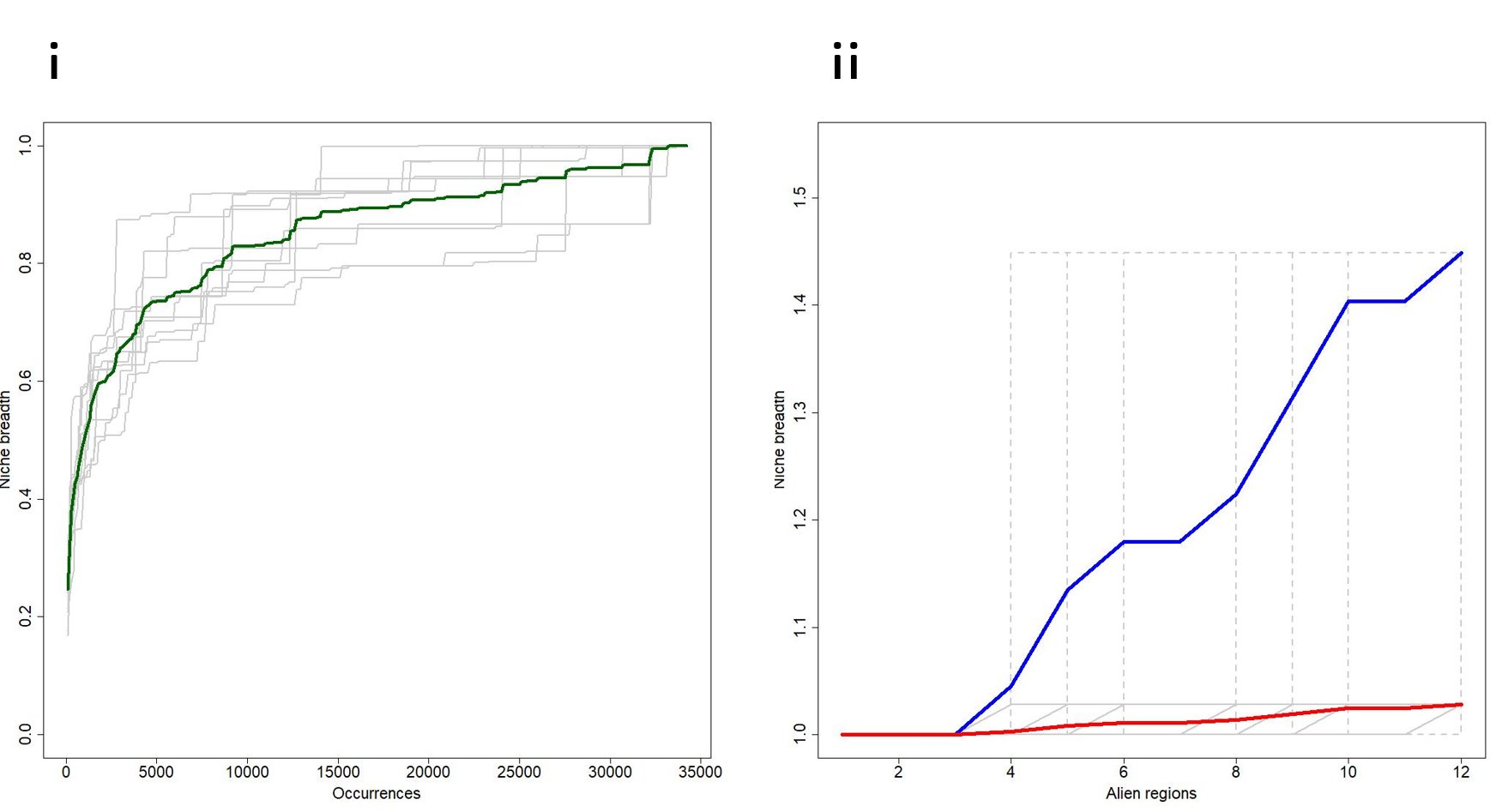


**I** - *Lepus europaeus* (Mammalia)


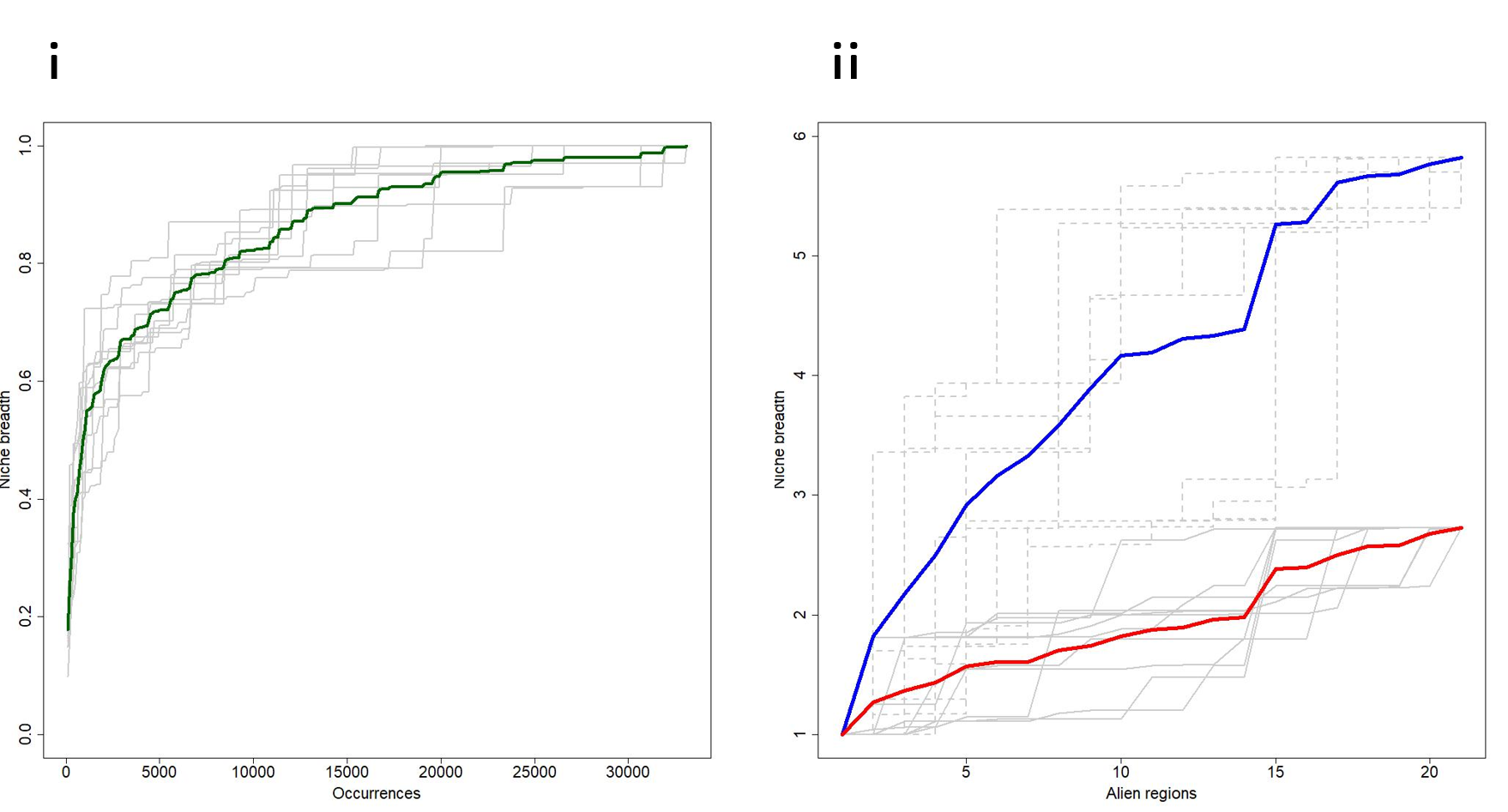


**J** - *Mustela nivalis* (Mammalia)


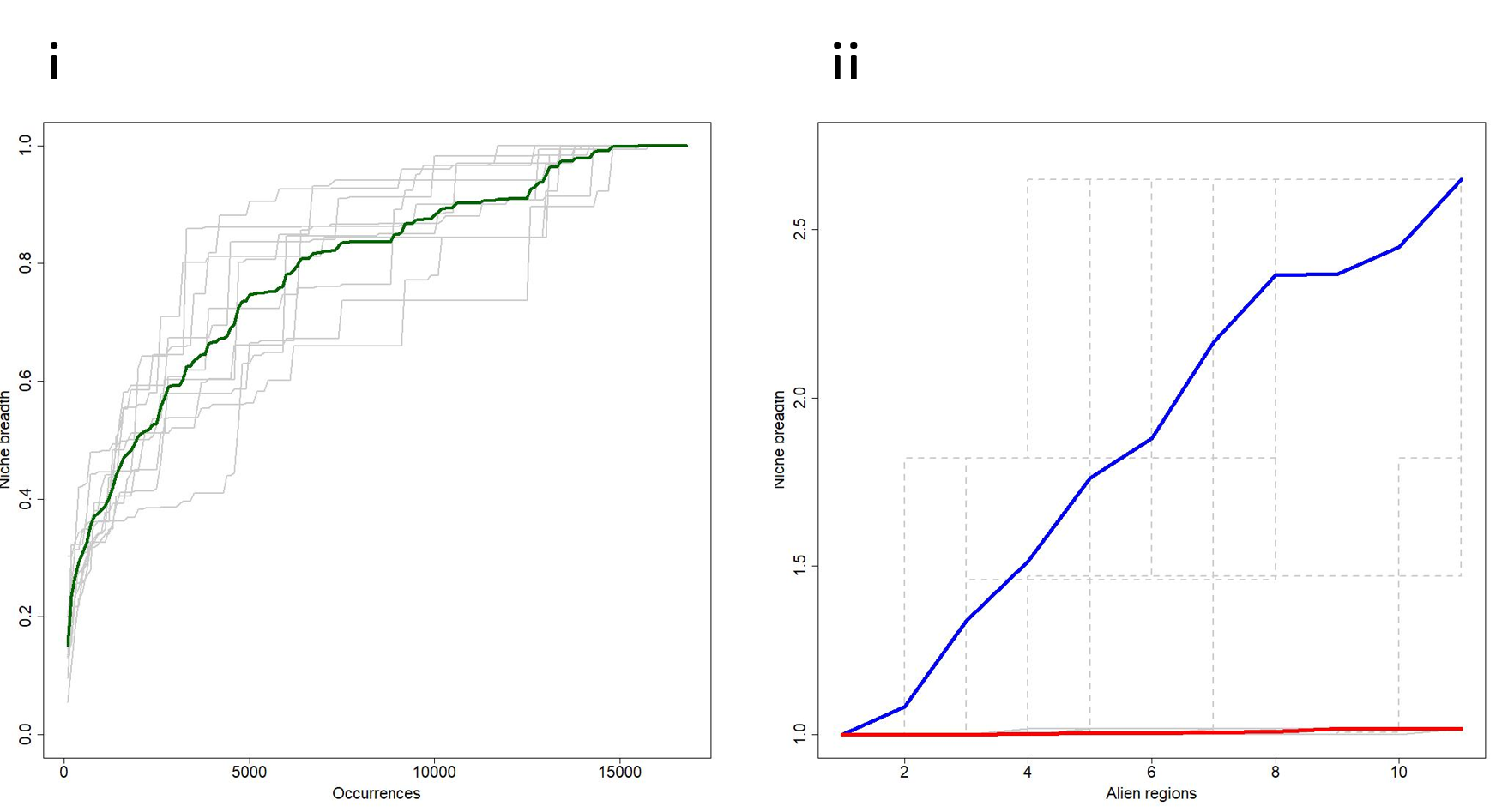


**K** - *Myocastor coypus* (Mammalia)


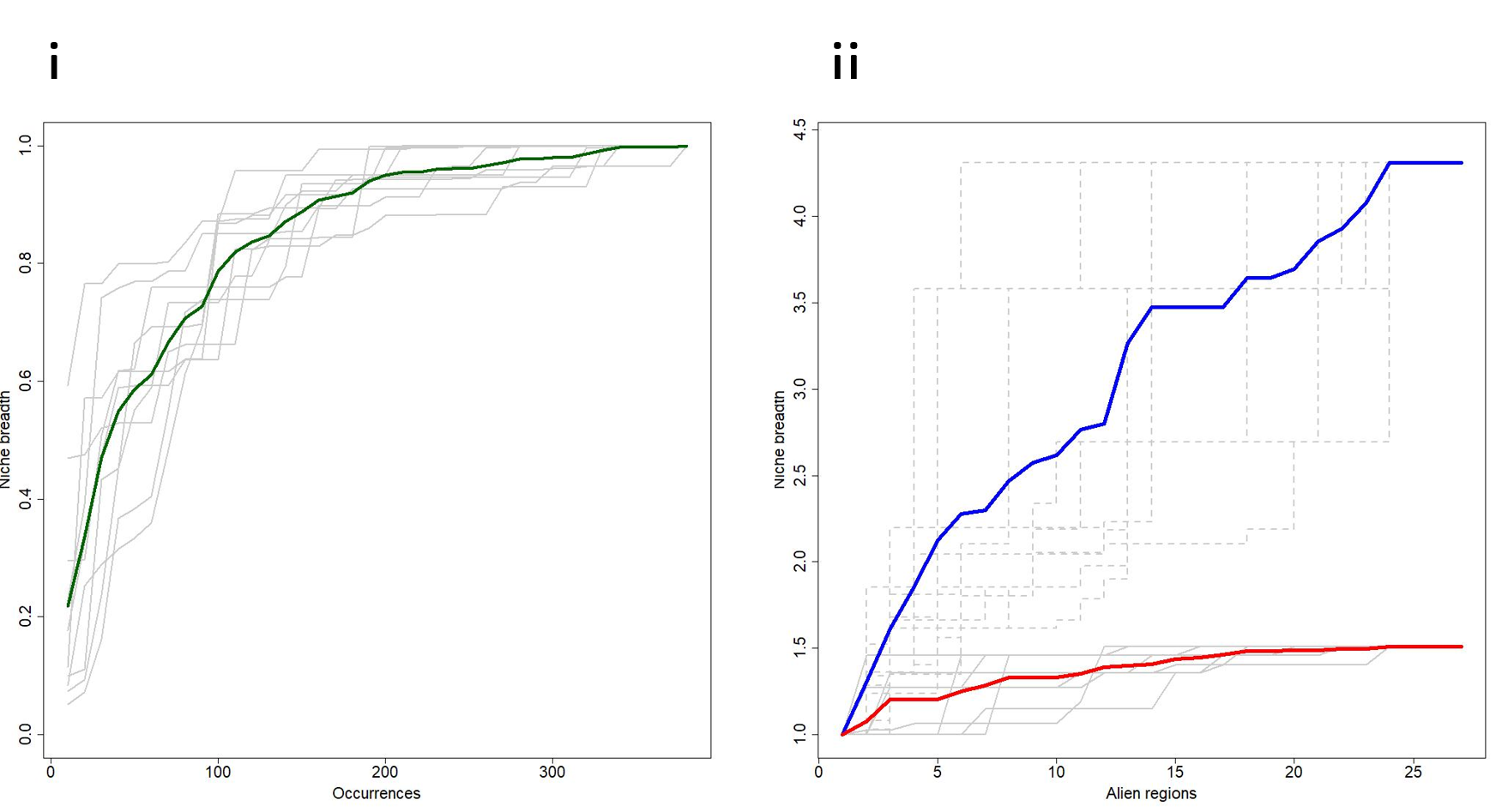


**L** - *Ondatra zibethicus* (Mammalia)


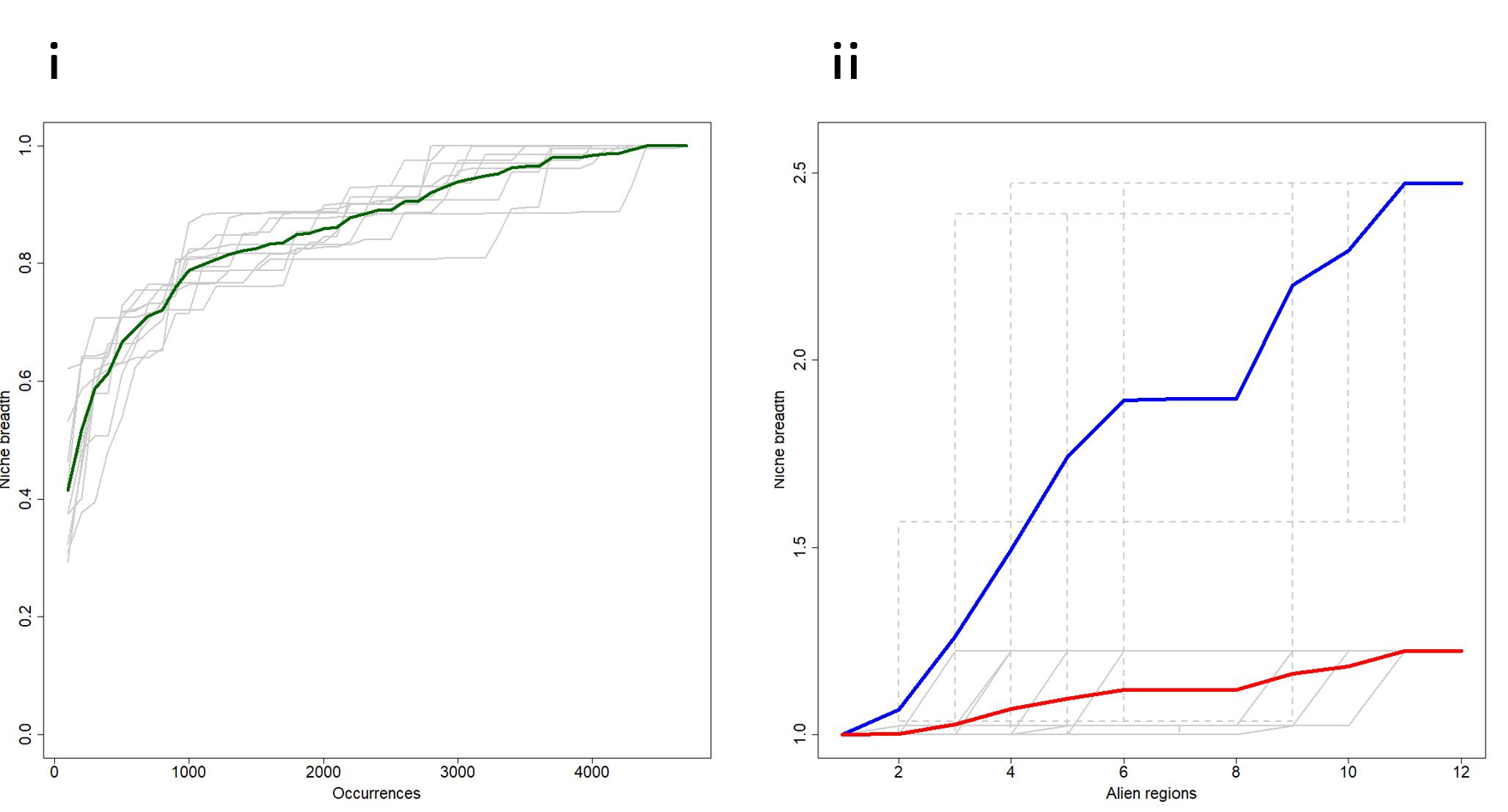


**M** - *Oryctolagus cuniculus* (Mammalia)


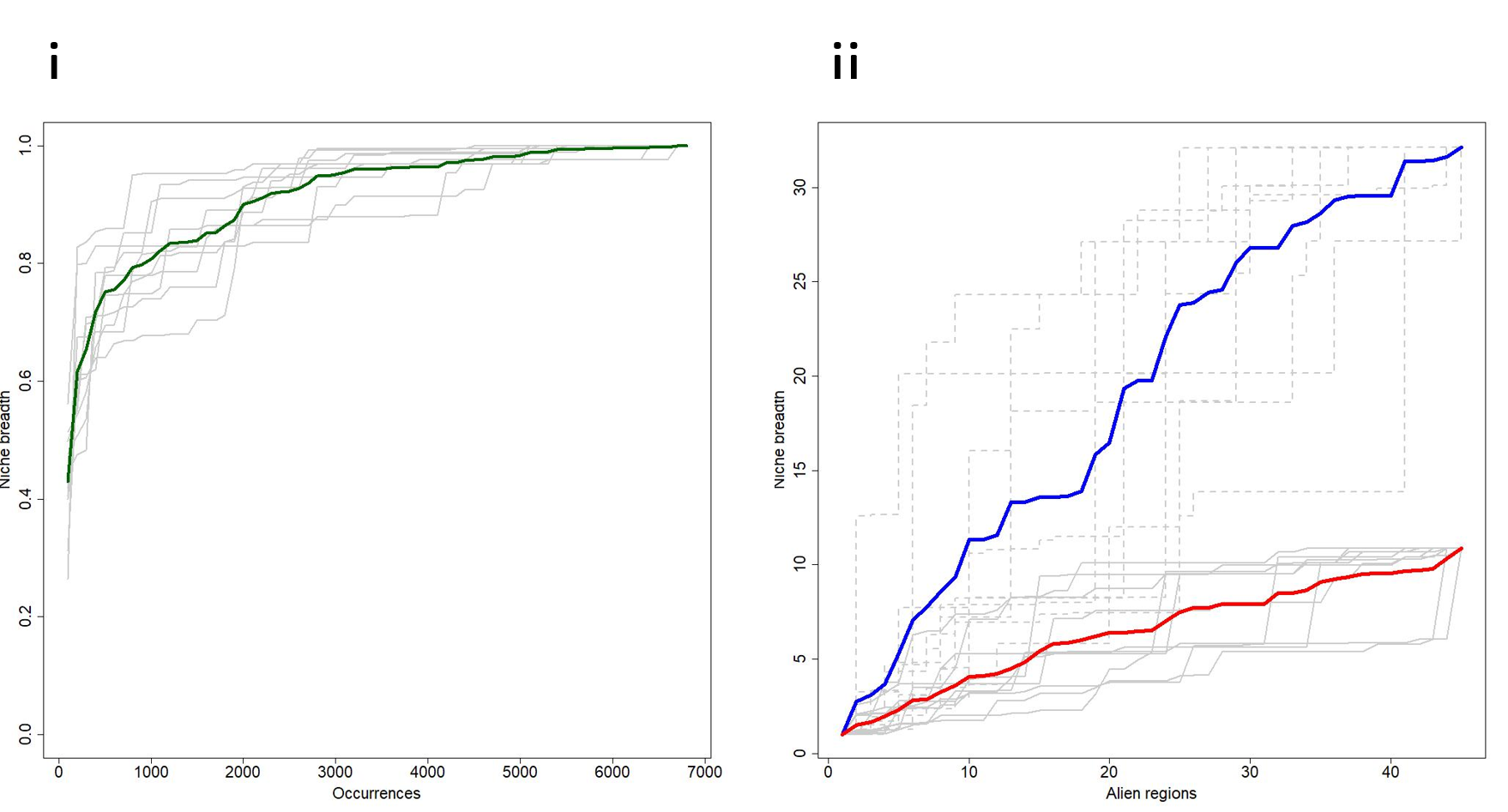


**N** - *Procyon lotor* (Mammalia)


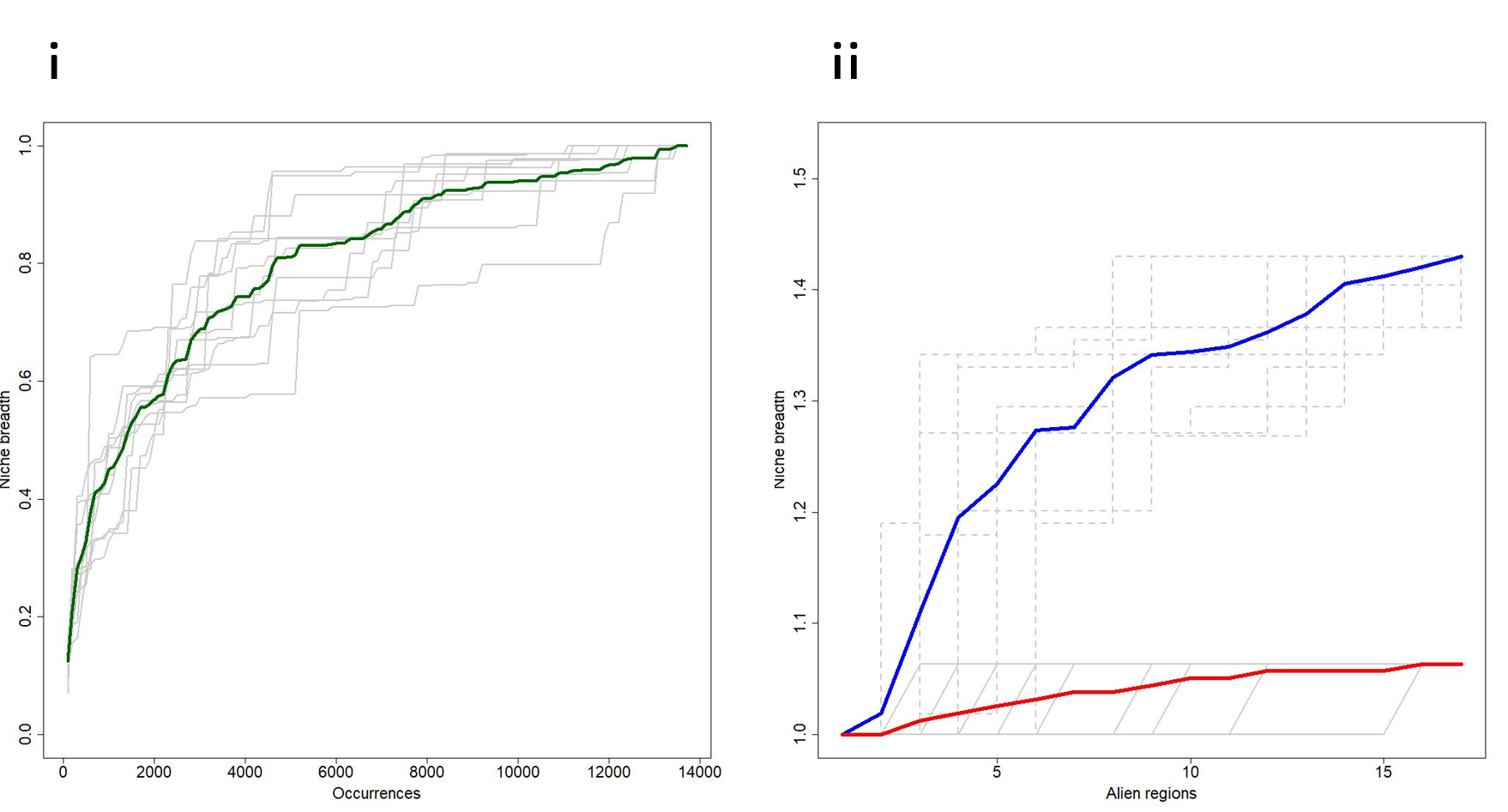


**O** - *Rattus tanezumi* (Mammalia)


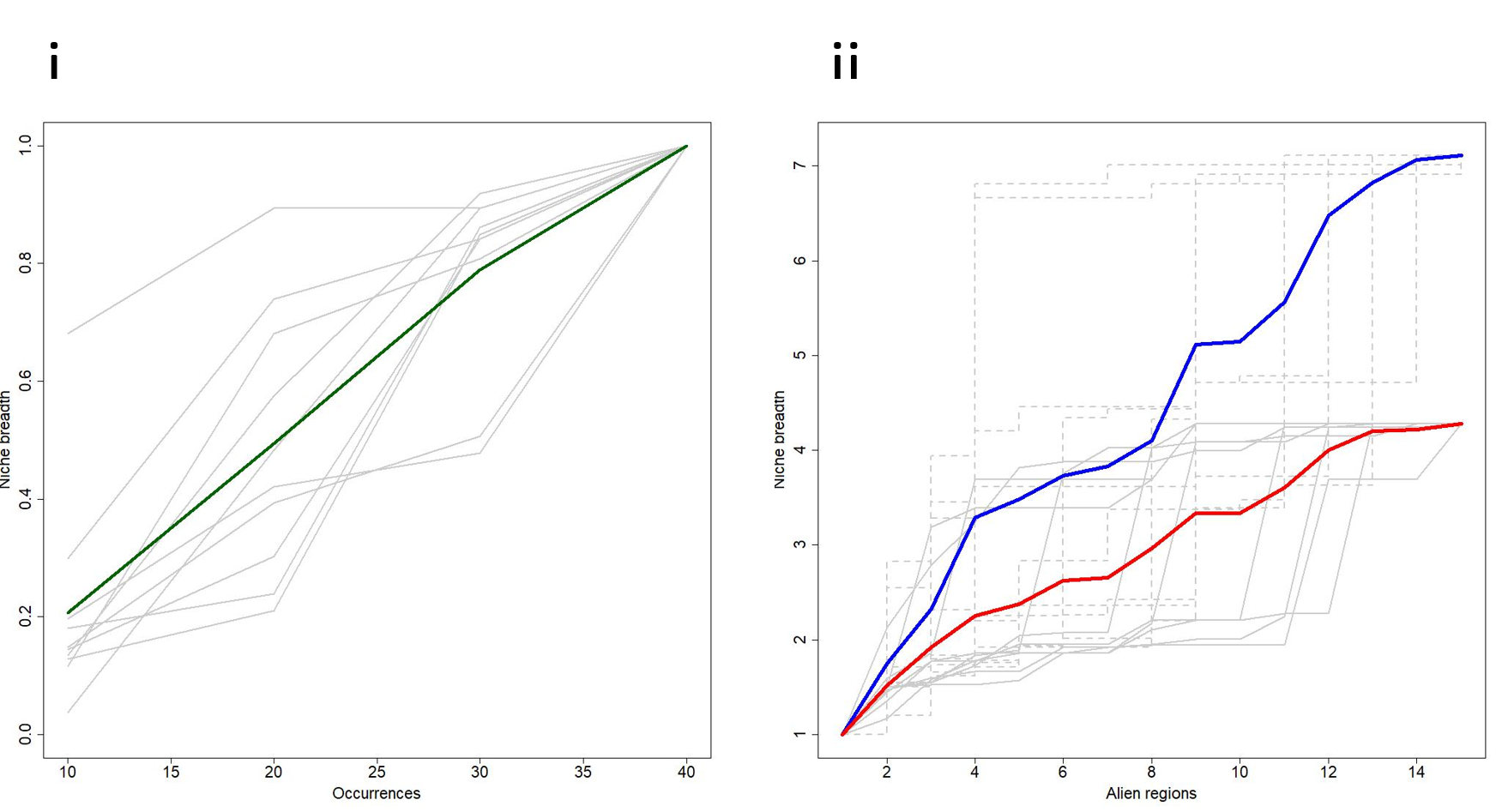


**P** - *Sciurus carolinensis* (Mammalia)


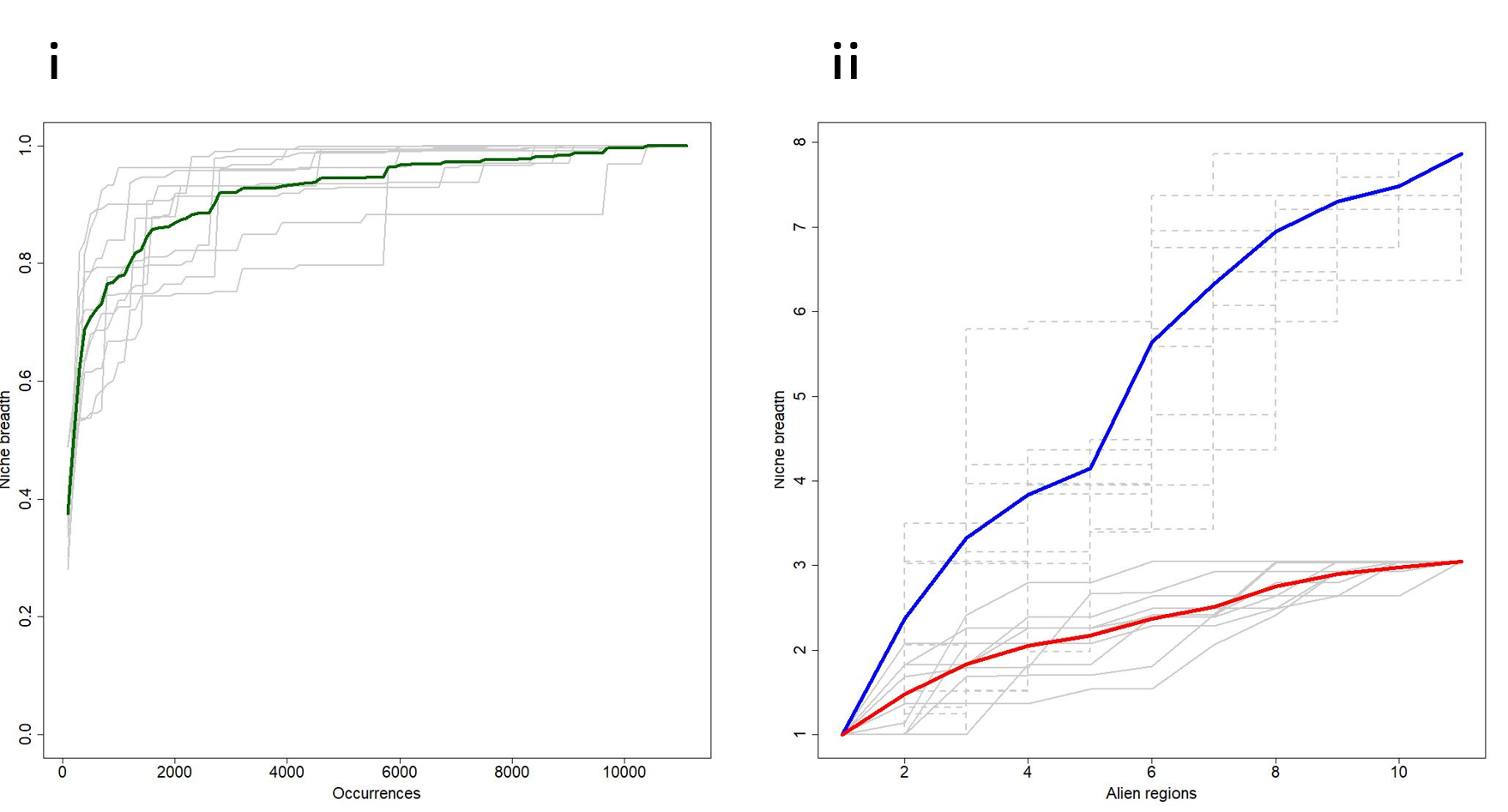


**Q** - *Vulpes vulpes* (Mammalia)


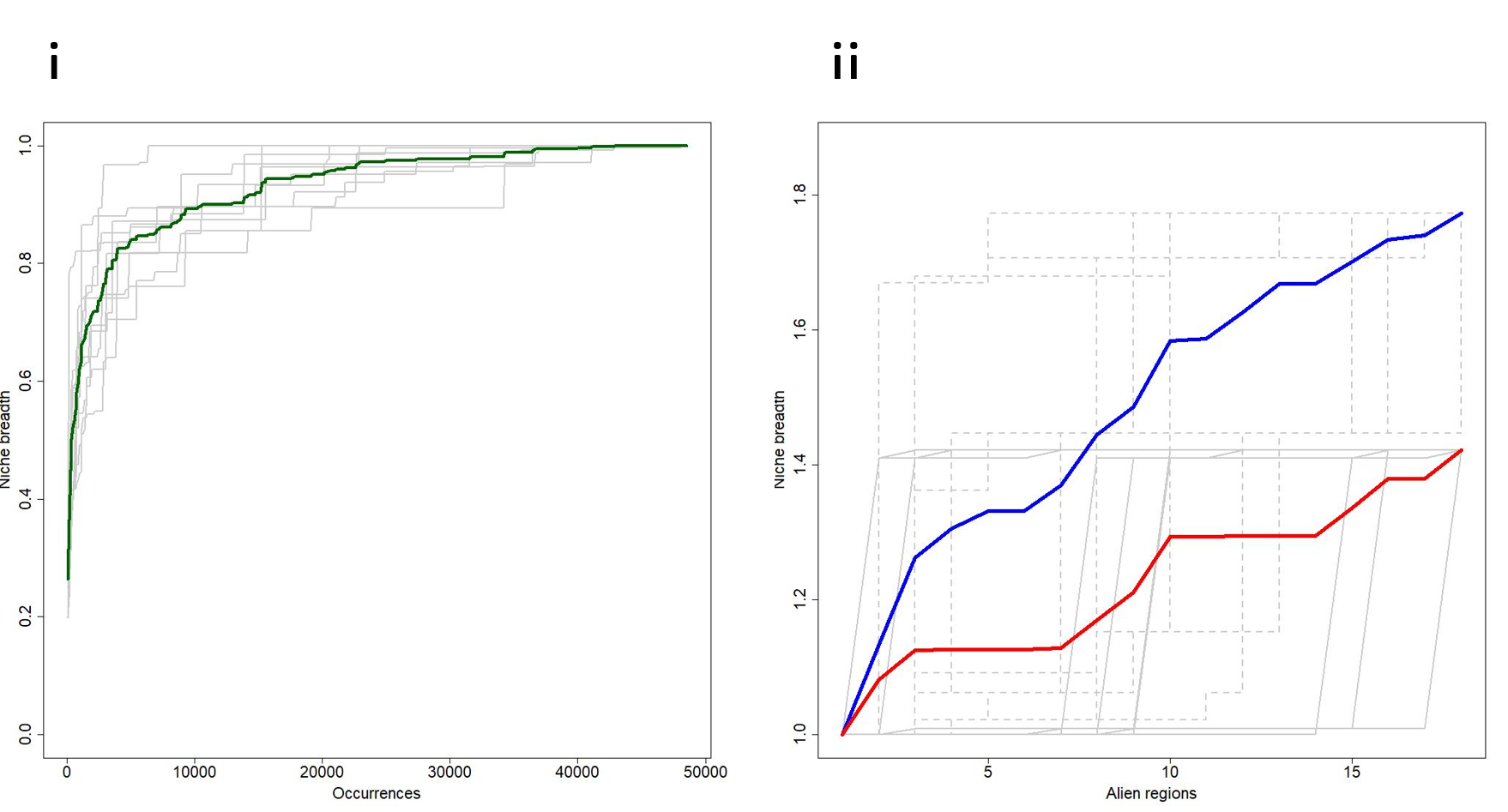


**R** - *Acridotheres tristis* (Aves)


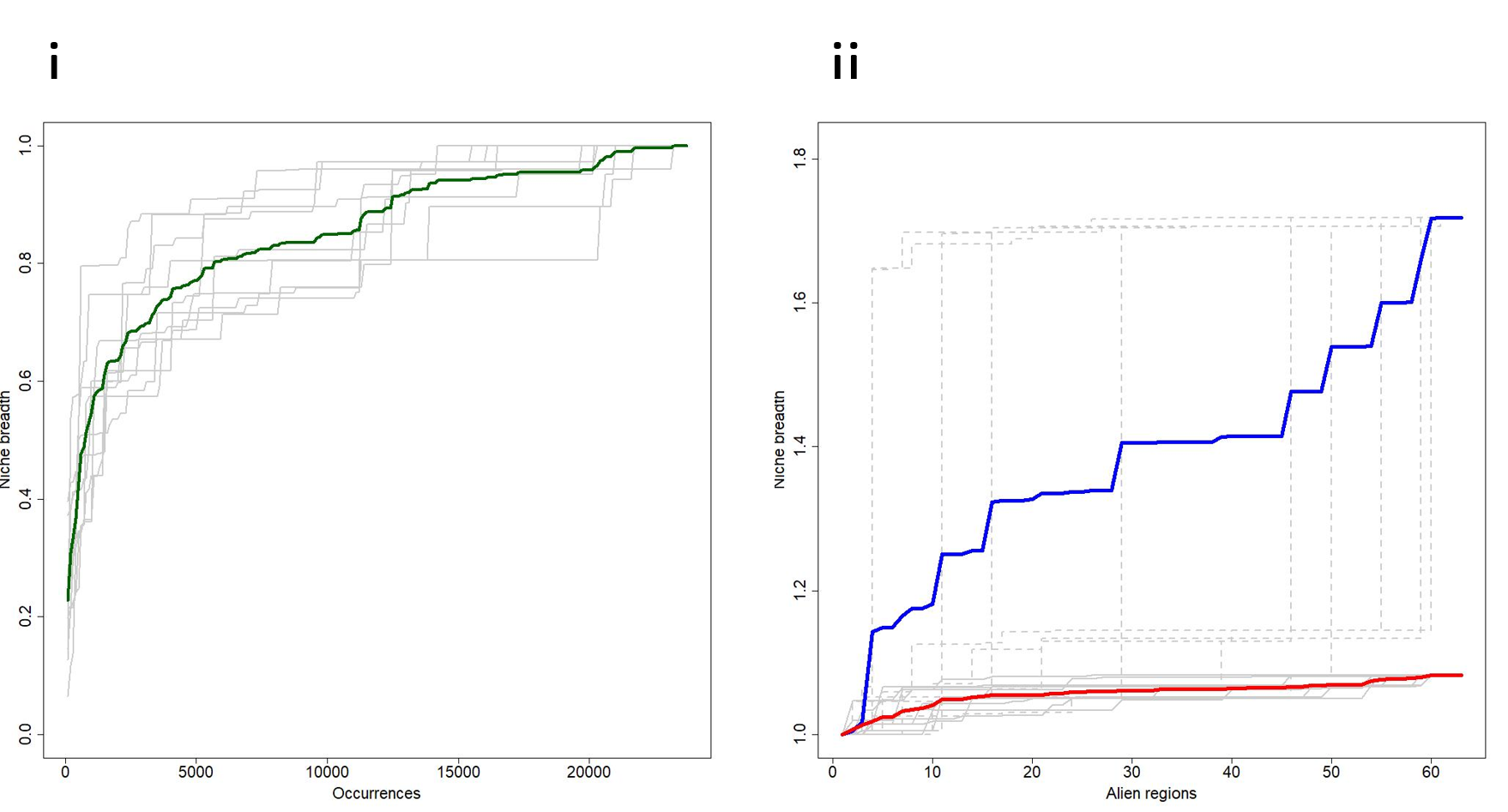


**S** - *Aix sponsa* (Aves)


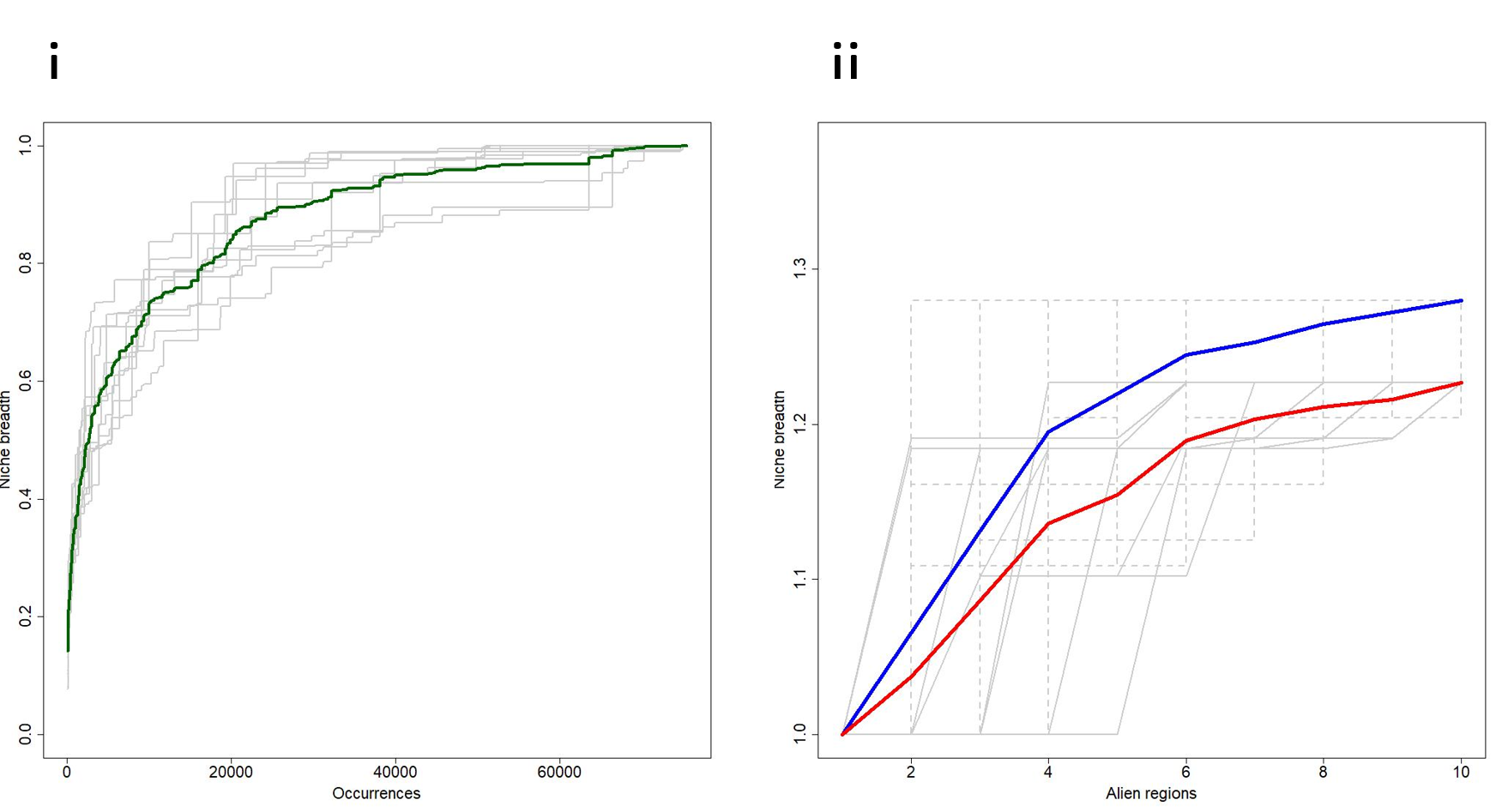


**T** - *Amandava amandava* (Aves)


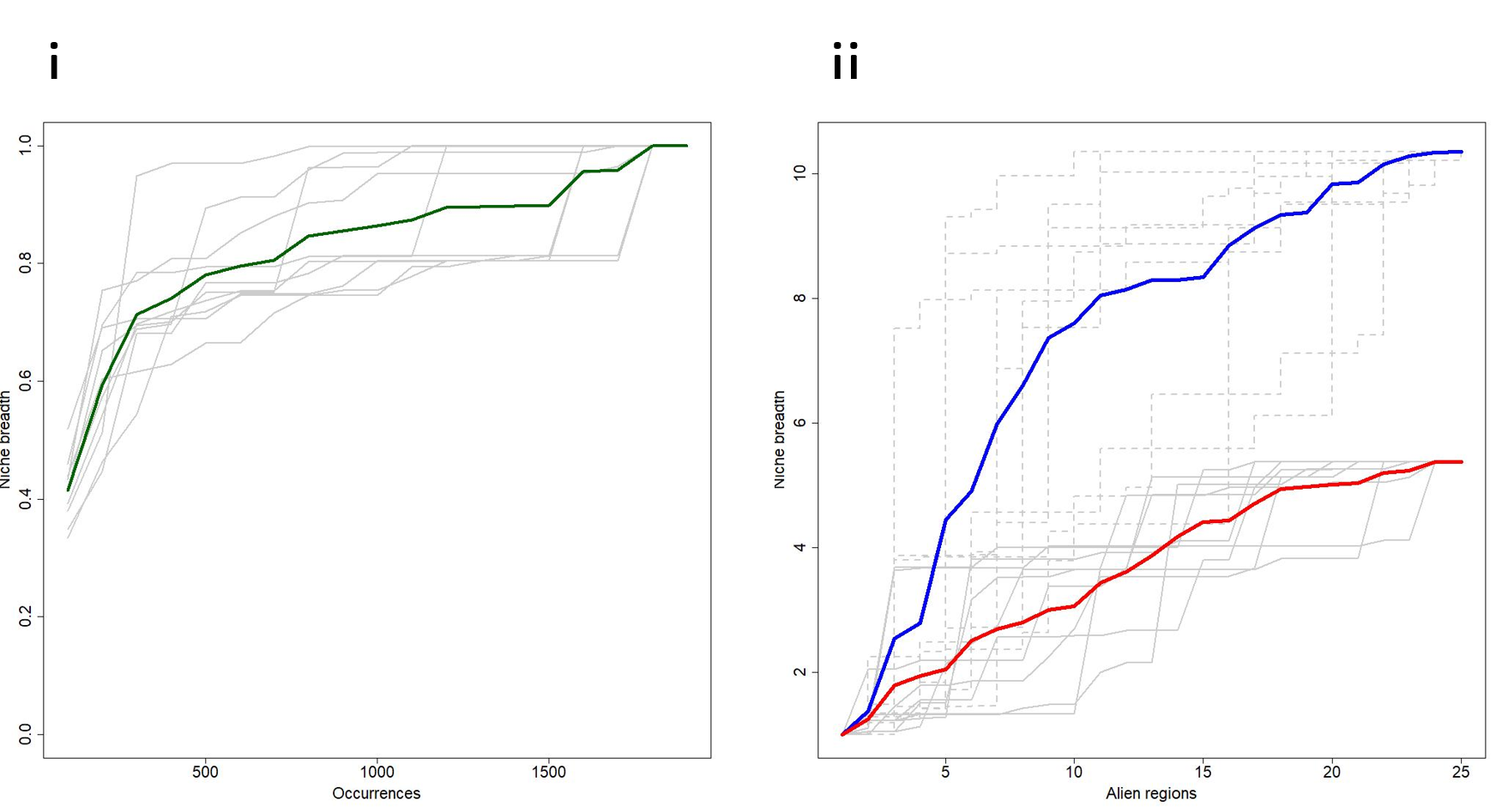


**U** - *Anas platyrhynchos* (Aves)


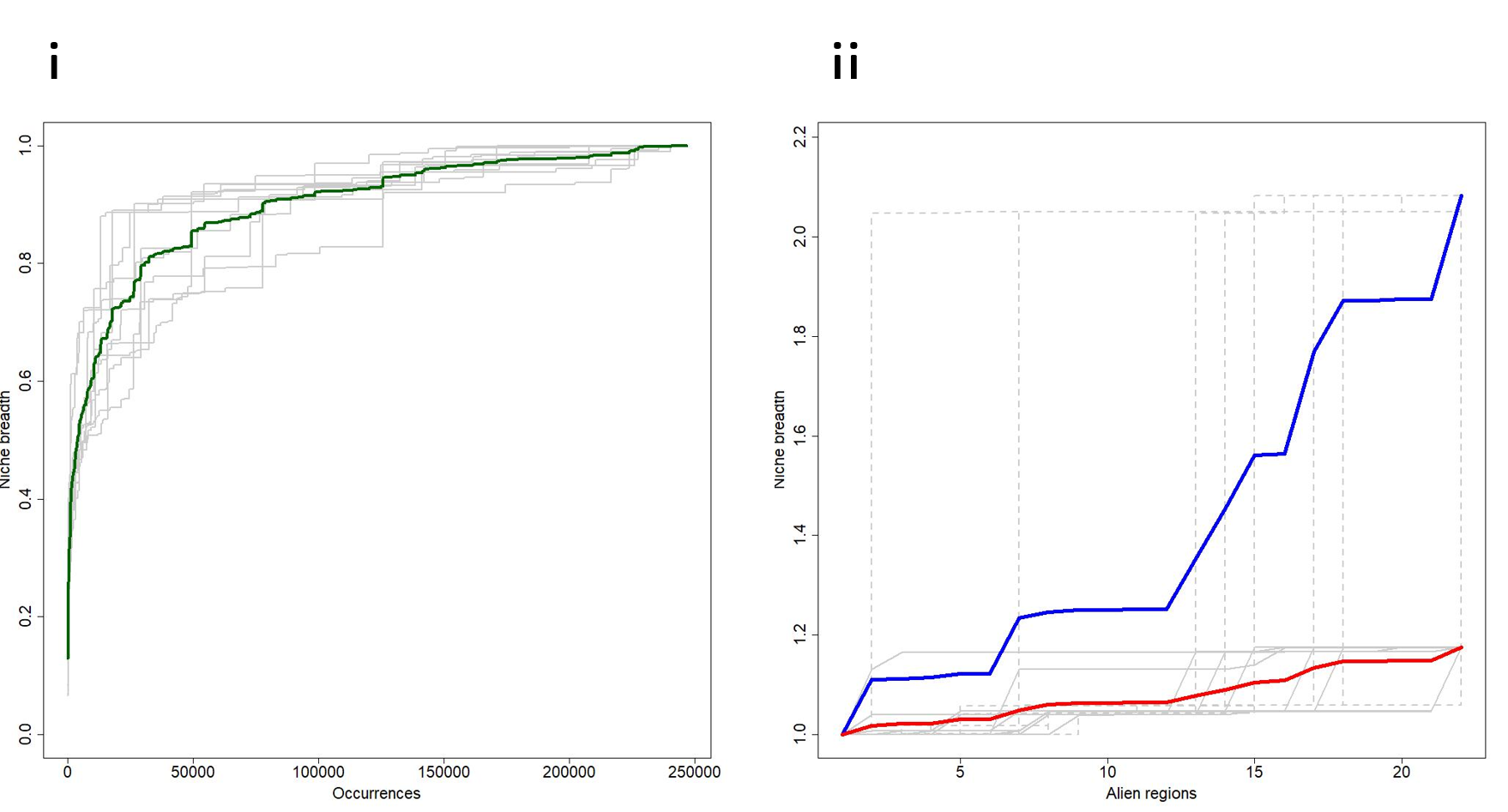


**V** - *Colinus virginianus* (Aves)


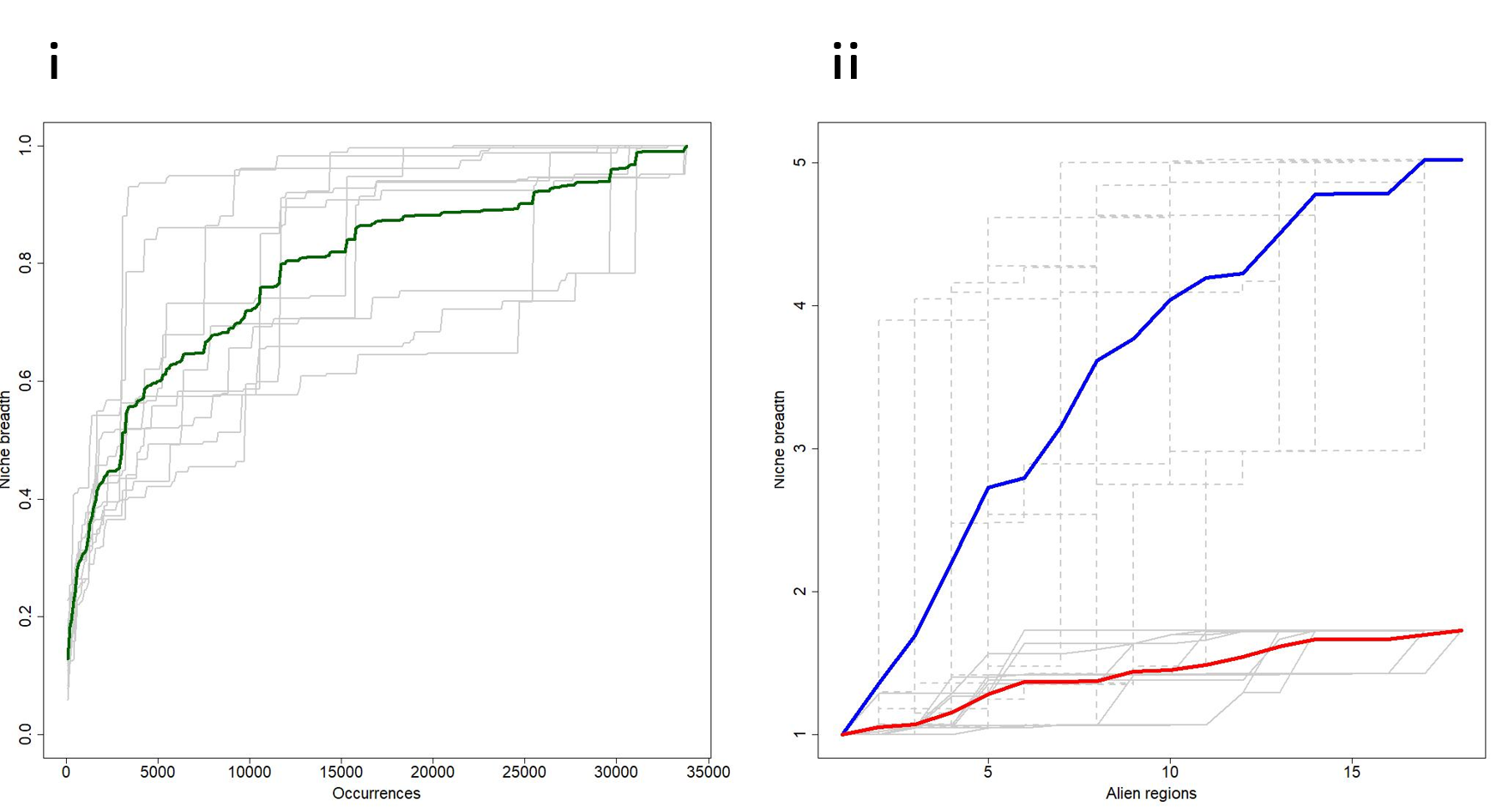


**W** - *Columba livia* (Aves)


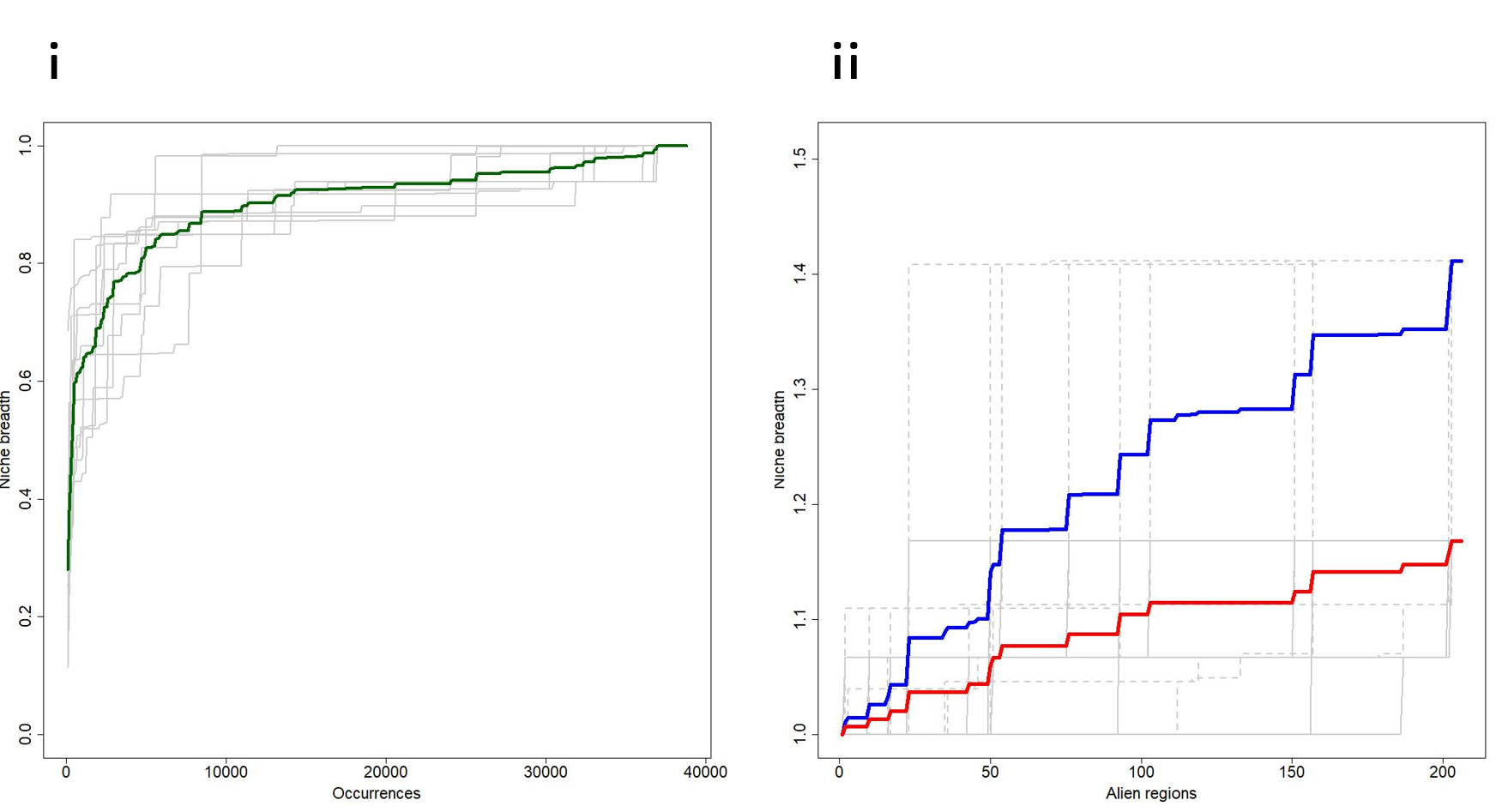


**X** - *Cygnus olor* (Aves)


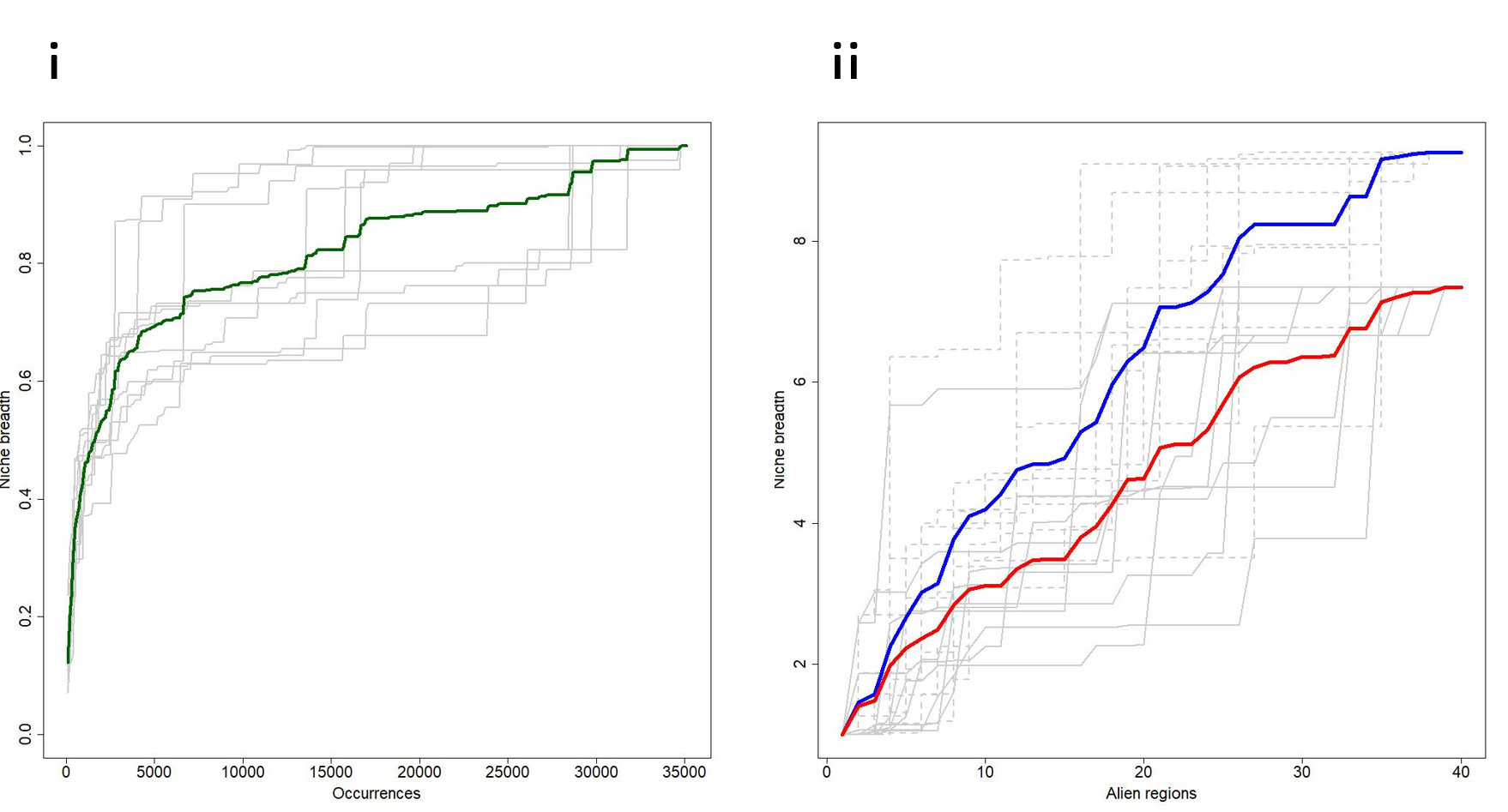


**Y** - *Gallus gallus* (Aves)


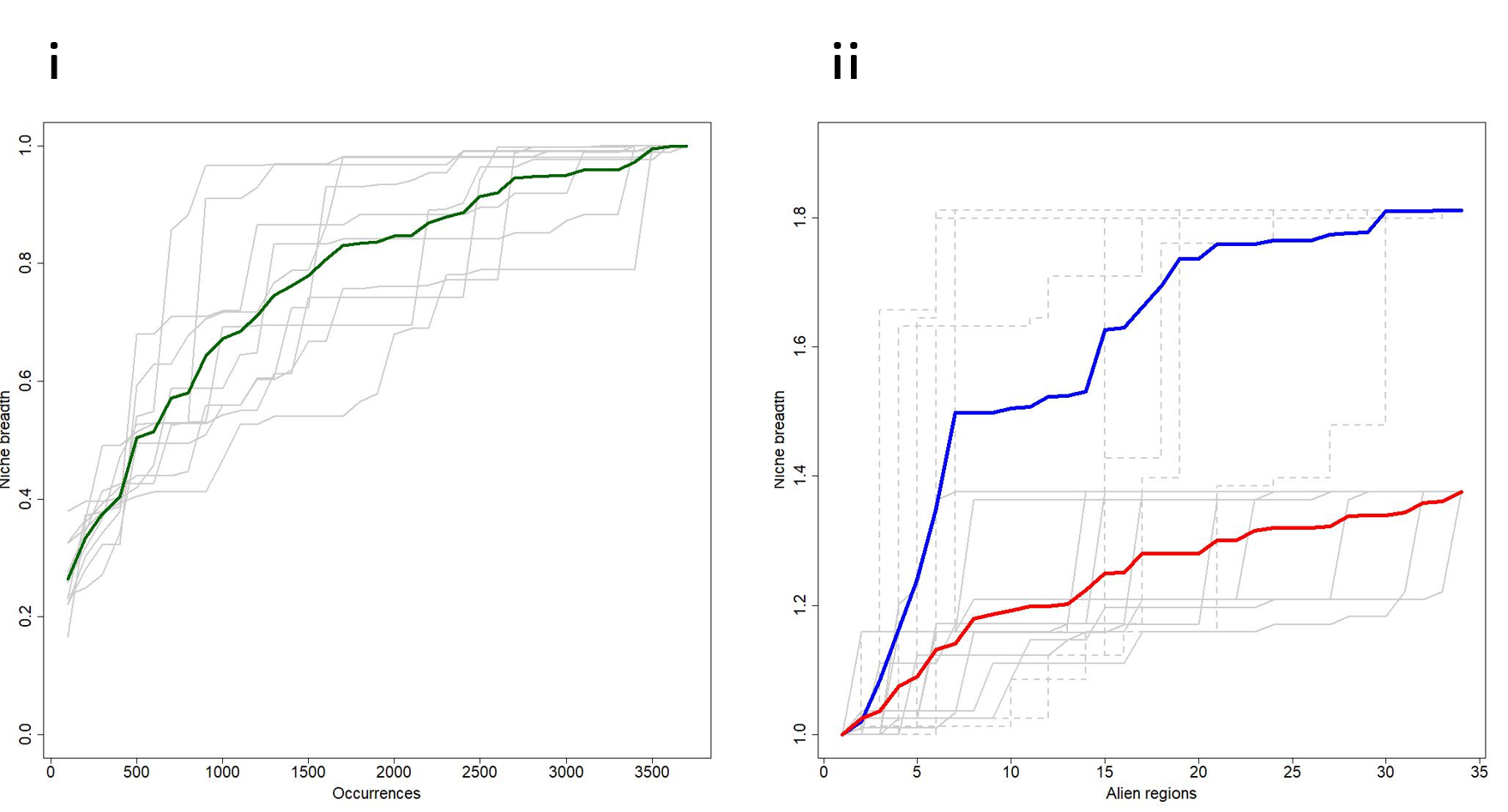


**Z** - *Geopelia striata* (Aves)


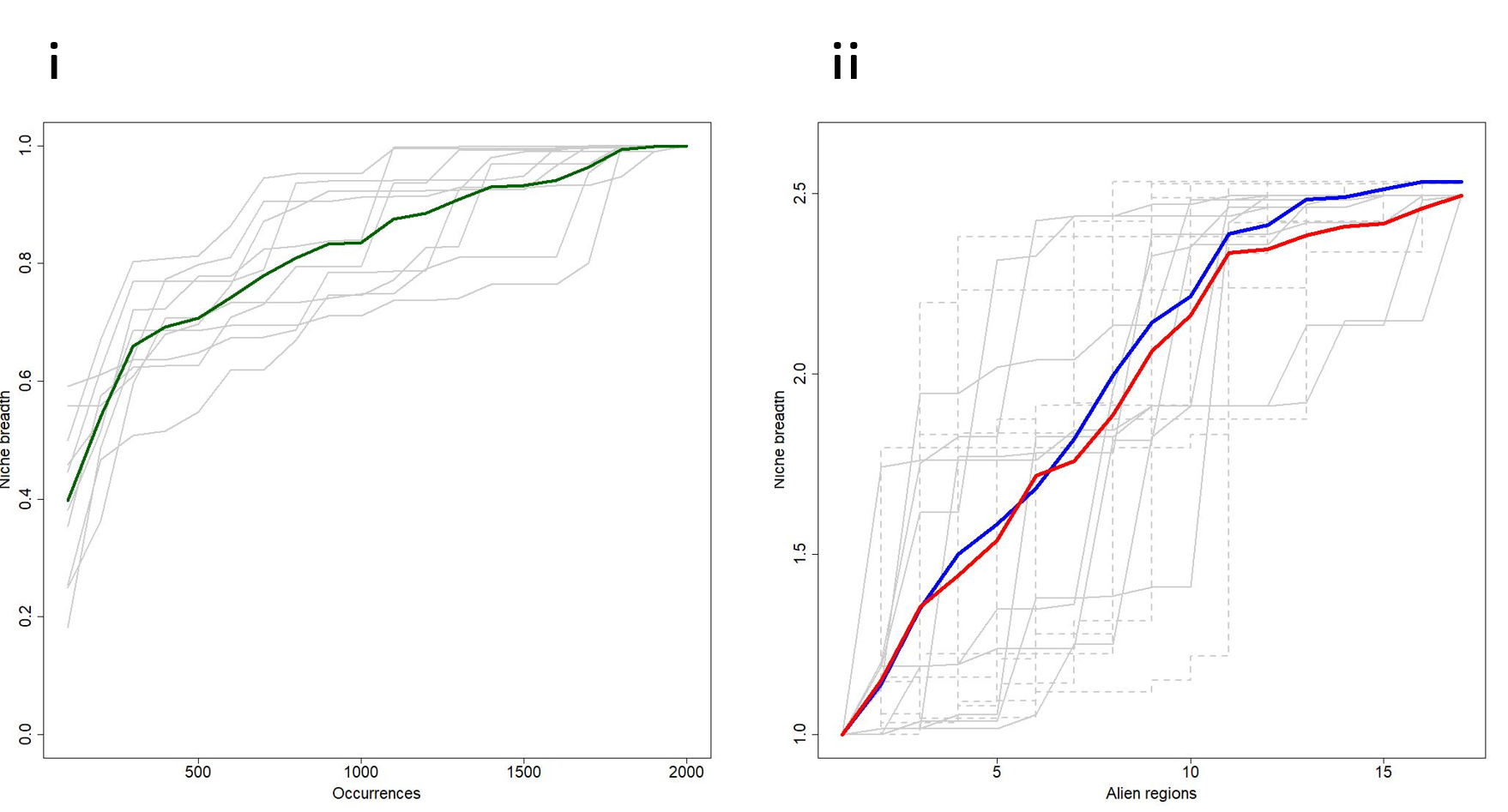

Supplement: Supplementary file 1 — Figure S1. [file ECE3-14-e11060-s001.docx]
